# Comparative Proteomics Reveals Strain-Specific β-TrCP Degradation via Rotavirus NSP1 Hijacking a Host Cullin-3-Rbx1 Complex

**DOI:** 10.1371/journal.ppat.1005929

**Published:** 2016-10-05

**Authors:** Siyuan Ding, Nancie Mooney, Bin Li, Marcus R. Kelly, Ningguo Feng, Alexander V. Loktev, Adrish Sen, John T. Patton, Peter K. Jackson, Harry B. Greenberg

**Affiliations:** 1 Department of Microbiology and Immunology, Stanford University School of Medicine, Stanford, California, United States of America; 2 Department of Medicine, Division of Gastroenterology and Hepatology, Stanford University School of Medicine, Stanford, California, United States of America; 3 Palo Alto Veterans Institute of Research, VA Palo Alto Health Care System, Palo Alto, California, United States of America; 4 Baxter Laboratory for Stem Cell Biology, Stanford University School of Medicine, Stanford, California, United States of America; 5 Institute of Veterinary Medicine, Jiangsu Academy of Agricultural Sciences, Nanjing, China; 6 Department of Veterinary Medicine, University of Maryland, College Park, Maryland, United States of America; University of Pittsburgh, UNITED STATES

## Abstract

Rotaviruses (RVs) are the leading cause of severe gastroenteritis in young children, accounting for half a million deaths annually worldwide. RV encodes non-structural protein 1 (NSP1), a well-characterized interferon (IFN) antagonist, which facilitates virus replication by mediating the degradation of host antiviral factors including IRF3 and β-TrCP. Here, we utilized six human and animal RV NSP1s as baits and performed tandem-affinity purification coupled with high-resolution mass spectrometry to comprehensively characterize NSP1-host protein interaction network. Multiple Cullin-RING ubiquitin ligase (CRL) complexes were identified. Importantly, inhibition of cullin-3 (Cul3) or RING-box protein 1 (Rbx1), by siRNA silencing or chemical perturbation, significantly impairs strain-specific NSP1-mediated β-TrCP degradation. Mechanistically, we demonstrate that NSP1 localizes to the Golgi with the host Cul3-Rbx1 CRL complex, which targets β-TrCP and NSP1 for co-destruction at the proteasome. Our study uncovers a novel mechanism that RV employs to promote β-TrCP turnover and provides molecular insights into virus-mediated innate immunity inhibition.

## Author Summary

Rotaviruses (RVs) are the leading cause of diarrhea in young children and lead to over 215,000 deaths annually worldwide. For virtually every mammal, there are RV strains specifically adapted to replicate efficiently in that host species. The success of RV, to a large extent, derives from its extraordinary ability to suppress the host antiviral responses in a host range restricted manner. One viral protein, named NSP1, is primarily responsible for this process. In this paper, using an unbiased approach, we discovered that NSP1’s ability to inhibit the host innate immune responses by promoting β-TrCP degradation is dependent on its specific interaction with a protein degradation machine called the Cullin-E3 ligase complex within the RV infected cell. Blocking this complex tremendously reduces NSP1’s ability to antagonize the host immune response and is detrimental for RV replication. Our study uncovers an unexpected role of Cullin-E3 complex in RV immune evasion and has broad implications for other viral pathogens.

## Introduction

β-transducin repeat-containing protein (β-TrCP, encoded by BTRC) is the core substrate recognition component of the Skp1-Cul1-F-box (SCF)^β-TrCP^ E3 ubiquitin ligase complex, which plays essential roles in a variety of biological processes, including apoptosis, cell cycle, carcinogenesis and innate immunity [1–3]. β-TrCP was originally discovered as an HIV-1 accessory protein Vpu-interacting protein that the virus hijacks for CD4 degradation to prevent super-infection [4]. β-TrCP recognizes a specific phosphorylated DSGX_(2+n)_S motif, known as a phosphodegron, present in its substrates such as β-catenin and IκBα, which are subsequently targeted for ubiquitin-mediated degradation [5,6]. In the canonical nuclear factor-κB (NF-κB) signaling cascade, pro-inflammatory cytokines or PRR (pattern recognition receptor) ligands stimulate rapid IκB phosphorylation, via the IKK complex, creating a phosphodegron recognized and ubiquitinated by the SCF^β-TrCP^ complex, and thus targeted for proteasomal degradation [7]. With the destruction of IκB, the otherwise sequestered NF-κB p65/p50 heterodimer is able to translocate from cytoplasm to the nucleus and activate a multitude of downstream genes, including chemokines and interferon (IFN) [8]. Therefore, β-TrCP is indispensable for an intact NF-κB signaling and optimal antiviral response. Despite its significance in modulating many biological processes, how β-TrCP itself is regulated remains largely unknown.

The leading cause of severe dehydration and often life-threatening diarrhea in children, rotaviruses (RVs) are responsible for an annual death rate of over 215,000 people worldwide, particularly in underdeveloped countries where current vaccines are significantly less effective [9,10]. RV is highly infectious and well known for its extraordinary ability to counteract host innate immunity [11,12], an effect primarily mediated by the non-structural protein 1 (NSP1) both *in vitro* and *in vivo* [13–15]. Interestingly, the ability of individual NSP1s derived from different RV isolates to counteract the innate immune response is highly host range restricted and displays a profound functional divergence in their mode of assisting virus replication. While NSP1s from multiple human and porcine RVs mediate the degradation of β-TrCP to inhibit the NF-κB pathway, NSP1s from other animal RV strains preferentially degrade interferon regulatory factor (IRF) proteins (i.e. IRF-3, 5, 7, 9) to block IFN production [16–19]. Traditionally, NSP1 has been categorized as a viral E3 ligase due to the presence of an N-terminal RING-finger domain [11,20]. However, biochemical evidence to corroborate this hypothesis is lacking. Recently, a phosphodegron-like (PDL) motif was identified in NSP1 of human and porcine RVs and this motif is required for inhibition of NF-κB signaling [21], potentially serving as a pseudo-substrate antagonist of β-TrCP [22]. The precise molecular mechanism by which NSP1’s targets are identified and degraded remains unclear.

Systematic identification of protein-protein interactions (PPIs) has proven instrumental for understanding how viruses usurp the host machinery to manipulate and repurpose signal transduction pathways. Recently, multiple viral pathogens, including HIV, HCV, influenza, and KSHV, have been successfully interrogated using such mapping strategies [23–26]. Here, using a comparative proteomics approach to examine the NSP1 “interactome”, we report that components of host Cullin-RING ubiquitin ligase (CRL) complexes, in particular, the cullin-3 (Cul3) CRL scaffold protein and the shared E3 ubiquitin ligase RING-box protein 1 (Rbx1), are essential for RV NSP1 mediated degradation of β-TrCP. NSP1, via its COPII sorting motif, localizes to the Golgi apparatus during RV infection and mediates the interaction between Cul3 CRL and β-TrCP with its N- and C-terminal domains respectively. NSP1 along with its substrate β-TrCP are subsequently coordinately degraded at the proteasome. Blocking the activity of Cul3 or Rbx1, by either small interfering RNA (siRNA) knockdown, chemical inhibition, or dominant-negative mutants, significantly impedes NSP1’s ability to down-regulate β-TrCP and is detrimental for RV replication. These findings demonstrate that RV NSP1, through re-directing the host Cul3-CRL complex, launches a suicide attack on β-TrCP, block NF-κB activation and thereby provide a permissive cellular environment for efficient viral propagation.

## Results

### NSP1 interactive network reveals strain-specificity in substrate recognition and strong interaction with the host Cullin-RING E3 ligase complex

**Fig 1:**
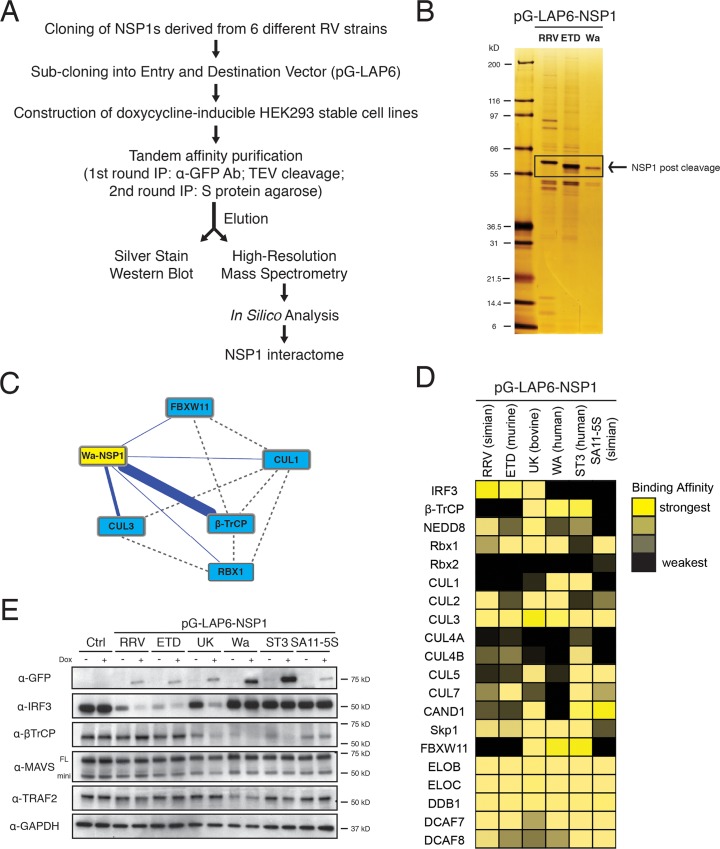
Proteomics analysis of rotavirus NSP1-host interactions. (A) Workflow diagram of affinity purification-mass spectrometry (AP-MS) pipeline used to identify NSP1-interacting host proteins. (B) Representative silver-stained SDS-PAGE of elutes (post TEV cleavage) from HEK293 cell lines stably expressing NSP1s derived from RV strains: RRV (simian), ETD (murine), or Wa (human). (C) Network representation of the bait protein Wa-NSP1 (yellow node) and some high-confidence host interaction partners (cyan nodes). Solid blue lines represent interactions identified in this study. Dotted grey lines indicate curated PPIs in public proteomics databases. (D) Heat map summary of a few host proteins (IRF3, β-TrCP, and CRL complex) interacting with different NSP1s (RRV and SA11-5S: simian RVs; WA and ST3: human RVs; UK: bovine RV; ETD: murine RV). The color corresponds to the number of peptides identified in the AP-MS experiments. (E) (Western blot (WB) of lysates using WT HEK293 cells and HEK293 stable cell lines, with or without doxycycline (dox) treatment, probed for indicated antibodies (FL: full-length MAVS; mini: mini-MAVS).

To systematically interrogate the complex molecular interactions between rotavirus and host, we recently employed a quantitative proteomics approach to construct a comprehensive interaction network map and identify host factors that interact with each of the twelve RV proteins. In this study, we focus specifically on the PPIs of NSP1, the pivotal RV virulence gene that assists virus replication through inhibition of the IRF and NF-κB signaling cascades [15]. To directly compare the differences between NSP1s derived from various RV strains, we adopted the G-LAP-Flp strategy, previously devised for mammalian proteomics studies [27], by fusing a LAP tag (EGFP-TEV-S-peptide) to the N-terminus of the selected NSP1 proteins and then generating doxycycline-inducible HEK293 stable cell lines expressing NSP1s from two human (WA, ST3) and four animal RV strains (two simian RRV, SA11-5S, one murine ETD and one bovine UK) (Fig 1A). Following tandem-affinity purification and TEV protease cleavage to remove the EGFP tag, we separated eluted proteins using SDS-PAGE and visualized both NSP1 itself and a multitude of bands corresponding to host proteins by silver staining (Fig 1B). By means of liquid chromatography mass spectrometry (LC-MS/MS) and rigorous bioinformatics analysis, we identified hundreds of host factors for each NSP1 and constructed maps of high-confidence PPIs (S1–S6 Tables and Fig 1C). The strength of the interactions was scored based on the absolute number of spectral counts observed and the percentage of peptide coverage (S1A Fig).

The PPI network revealed several interesting findings. First, we noted that the two best characterized NSP1 substrates for degradation, IRF3 and β-TrCP, bind strongly to NSP1 in a strain-specific manner (Fig 1D). NSP1s from simian (RRV) and murine (ETD) RV strains exclusively bind to IRF3 while those from the two human (Wa, ST3) strains bind to β-TrCP. Interestingly, the bovine (UK) strain NSP1 interacts with both substrates, likely representing an evolutionarily intermediate viral protein. Another simian RV strain (SA11-5S) encodes a naturally occurring defective NSP1 mutant due to a C-terminal 17 amino acid truncation and it serves as the negative control that binds to neither IRF3 nor β-TrCP. Importantly, using the HEK293 stable expression cell lines and a transient transfection strategy, we confirmed that the protein degradation pattern perfectly matched the interaction data (Fig 1E and S1B Fig). Therefore, a functional dichotomy is present in human versus animal NSP1s’ ability in substrate binding and subsequent degradation of either IRF3 or β-TrCP. Notably, two other previously reported NSP1 substrates, MAVS and TRAF2 [28,29], were not identified in our interaction data and not observed to be degraded upon doxycycline induction (Fig 1E).

Second, we observed interactions between NSP1 and a host of proteins belonging to the CRL complexes, including Cullins 1–7, the shared E3 ligase subunit Rbx1 and other CRL-associated components and regulatory factors (Fig 1D). Cul3 has been previously observed to interact with porcine OSU-NSP1 [30] and more recently with NSP1s from several human and animal RV strains [31]. Our proteomics data has allowed us to not only confirm the Cul3 binding of NSP1 but also expand to the breadth of the analysis to multiple other cullin members and associated proteins. Similar to β-TrCP, Cul1 and FBXW11 specifically interact with Wa, ST3 and UK NSP1s whereas other proteins, including Cul2, 3, 5 and Rbx1 are broadly reactive with all the NSP1s. CRLs are multi-protein complexes responsible for targeting many substrates for degradation both in yeast and higher eukaryotes [32]. It is precisely because of their significance that CRL complexes are frequently rewired by viral pathogens for immune evasion purposes [33]. Recently, HBV X protein was reported to hijack a Cul4-DDB1 CRL to target the Smc5/6 complex for degradation to allow productive HBV gene expression [34]. Therefore, we hypothesized that in the case of NSP1, certain CRLs were being appropriated by specific RV strains to favor their replication in a host range restricted fashion. The expansive interaction of several NSP1s with Rbx1 raised an important question as to whether NSP1, by itself, functions as the purported viral E3 ligase or may redirect CRLs towards specific targets.

Third, our data provided quantitative profiling of host E2 ubiquitin-conjugating enzymes that co-precipitated with different NSP1s (S1C Fig). Of note, the *in vitro* reconstitution of ubiquitin ligase activity using recombinant NSP1 has not been reported, which could be due to the lack of the proper E2 proteins. Several of these E2 enzymes are present at very low abundance [35] and might only be revealed by a large-scale proteomics survey such as this study.

Finally, we detected several previously unreported NSP1-interacting host proteins (S1–S6 Tables), exemplified by the CCT complex, which assists protein folding by acting as chaperone [36], AIFM1, an apoptosis-inducing factor [37], and TRIM28, a transcription factor that is also known as KAP1 and regulates the DNA damage response [38]. These newly identified NSP1 binding partners may unveil potentially novel regulatory functions of NSP1 other than modulating the innate immune response and will be examined in subsequent studies.

### Rbx1, Cul1, and Cul3 are required for NSP1-mediated β-TrCP degradation

**Fig 2:**
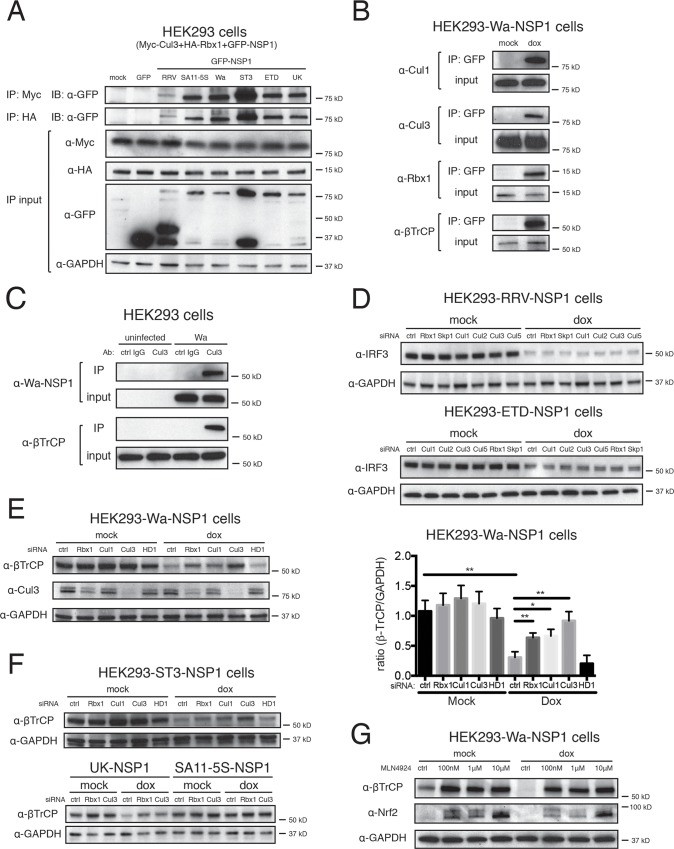
Cul3 and Rbx1 interact with NSP1 and contribute to β-TrCP degradation. (A) Lysates of HEK293 cells co-transfected with GFP-NSP1, Myc-Cul3, and HA-Rbx1 were subject to immunoprecipitation using α-Myc or α-HA antibody and probed for α-GFP antibody. (B) HEK293-Wa-NSP1 stable cell lines were treated with 1 μg/ml of doxycycline for 24 hr and harvested for immunoprecipitation using α-GFP antibody and probed for indicated antibodies. (C) Lysates of mock or Wa infected HEK293 cells (MOI = 3, 24 hpi) were subject to immunoprecipitation using control rabbit polyclonal or α-Cul3 antibody, and probed for indicated antibodies. (D) HEK293 cells stably expressing RRV or ETD-NSP1 were transfected with indicated siRNA, treated with doxycycline, and harvested for western blot using indicated antibodies. (E) Same experiment as in (D) except that HEK293 cells stably expressing Wa-NSP1 were used instead (HD1: HECTD1). Blots were quantified and the level of β-TrCP is normalized to loading control GAPDH. The ratio in mock-treated cells is set to 1. (F) Same experiment as in (D) except that ST3, UK, or SA11-5S NSP1s were used (HD1: HECTD1). (G) HEK293 cells stably expressing Wa-NSP1 were treated with MLN4924 and doxycycline, harvested for western blot using indicated antibodies. In all figures, experiments were repeated at least three times. Data are represented as mean ± SEM. Statistical significance is determined by Student’s t test (*p≤0.05; **p≤0.01; ***p≤0.001).

Based on the NSP1 interactive networks, we next set out to mechanistically characterize one of the highly enriched interactions/biological pathways, the CRL complex. We first validated the initially observed interactions (Fig 1D) between NSP1 and Rbx1, Cul1, Cul3 and β-TrCP by co-immunoprecipitation (IP) of transiently overexpressed NSP1s, Rbx1 and Cul3 and in the context of virus infection (Fig 2A–2C). Consistent with the high-throughput MS data, NSP1s from all RV strains co-purified with exogenously expressed Myc-tagged Cul3 and HA-tagged Rbx1 (Fig 2A). In addition, in pull-down experiments using lysates from HEK293 cells stably expressing Wa-NSP1, we observed that NSP1 co-precipitated with endogenous Cul1, Cul3, Rbx1 and β-TrCP (Fig 2B). Importantly, during Wa RV infection, we demonstrated that the natively expressed untagged Wa-NSP1 also co-precipitated with endogenous Cul3 (Fig 2C). It is noteworthy that Cul3 and β-TrCP only bound to one another during Wa RV infection, consistent with our previous knowledge of no reported interaction in the Wa-NSP1 network (Fig 1C).

After confirming the biochemical association between CRL components and NSP1, we began to assess their functional role using targeted siRNA knockdown. Although silencing of Rbx1, Skp1 and multiple Cullin members (Cul1, 2, 3, 5) did not affect IRF3 degradation by NSP1s derived from RRV and ETD strains (Fig 2D and S2A Fig), depletion of Rbx1, Cul1 or Cul3 by siRNA led to impaired β-TrCP degradation by Wa-NSP1 (Fig 2E and S3A Fig). In contrast, despite Cul2 and Cul5 binding to Wa-NSP1 (Fig 1D), specific siRNA against these two cullins, as well as those targeting Cul4A, 4B, 7, and an irrelevant E3 ligase HECTD1 did not affect β-TrCP reduction by Wa-NSP1 (Fig 2E, S2B and S2C Fig, and S3A and S3B Fig). These results highlight the specificity of Rbx1, Cul1, and Cul3 for regulating β-TrCP turnover. Moreover, we examined NSP1s from ST3 and UK strains, also capable of inducing β-TrCP degradation (Fig 1E). The reduced β-TrCP levels were significantly restored in both Rbx1-knockdown and Cul3-knockdown cells (Fig 2F), suggesting a common regulatory mechanism of β-TrCP. To exclude the possibility that our observation is cell type-dependent, we also tested the efficacy of Cul3 siRNA on preventing β-TrCP degradation in MA104 cells, an African green monkey cell line commonly used for RV propagation. Consistently, simian β-TrCP down-regulation induced by human RV Wa strain infection was counteracted by Cul3 depletion (S2D Fig).

In addition to siRNA silencing, we attempted to completely knock out Cul3 and Rbx1 in HEK293 cells via CRISPR-Cas9 genome editing. However, screening of several hundred colonies using three independent sgRNA sequences yielded only partial depletion of either gene, strongly suggesting the requirement of Cul3 and Rbx1 for cell survival, consistent with the reported phenotype of early embryonic lethality for both Cul3^-/-^ and Rbx1^-/-^ mice [39,40] and recently published gene essentiality list [41]. Nevertheless, even though Cul3 was not completely depleted, significant reduction in endogenous Cul3 levels led to pronounced inhibition of β-TrCP degradation mediated by Wa and ST3-NSP1 (S2E Fig).

As an alternative to confirm the function of Cul1, Cul3 and Rbx1 in promoting β-TrCP degradation, we treated Wa-NSP1 expressing cells with MLN4924, a small-molecule inhibitor that inactivates the NEDD8-activating enzyme, which is critical for the catalytic cycle of all known Cullins [42]. MLN4924 treatment efficiently blocked Wa/ST3-NSP1-mediated β-TrCP degradation at inhibitory concentrations (Fig 2G), paralleling the results with siRNA knockdown of Rbx1 and Cul3. The levels of Nrf2, a well-defined substrate of Cul3/Keap1/Rbx1 complex [43], also increased upon inhibitor treatment (Fig 2G). Taken together, our results strongly support the conclusion that the participation of host proteins Cul1, Cul3, and Rbx1 is required for NSP1-mediated degradation of β-TrCP.

### Depletion of Cul3 restores the NF-κB pathway and inhibits RV replication

**Fig 3:**
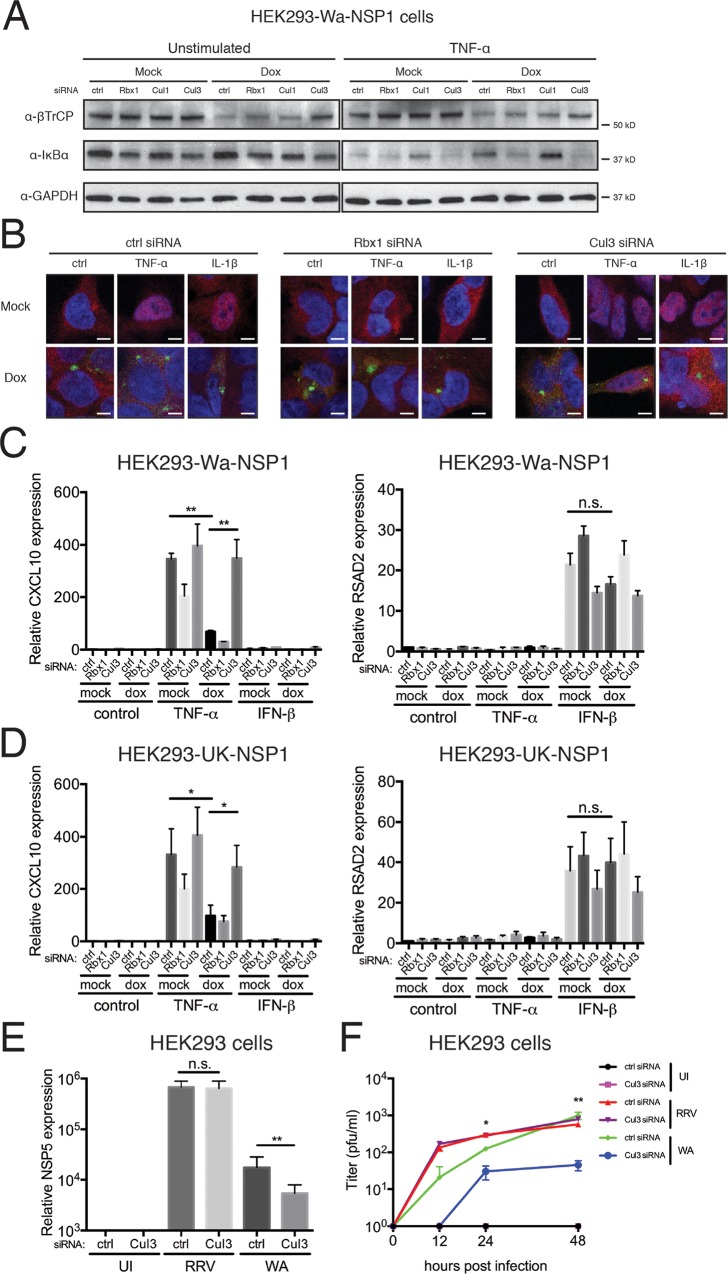
Cul3 attenuation restores IκB degradation and chemokine expression. (A) HEK293 cells stably expressing Wa-NSP1 were transfected with indicated siRNA, treated with doxycycline, and stimulated with TNF-α (10 ng/ml) for 15 min. Lysates were harvested for western blot using indicated antibodies. (B) Same experiment as in (A) except that cells were also stimulated with IL-1β (10 ng/ml) for 15 min and examined for the localization of NF-κB p65 subunit (red), Wa-NSP1 (green) and nucleus (DAPI, blue) by confocal microscopy. Panels are single z slices with a scale bar of 5 μm. (C) HEK293 cells stably expressing Wa-NSP1 were transfected with indicated siRNA, treated with doxycycline, and stimulated with TNF-α (10 ng/ml) or IFN-β (100 U/ml) for 6 hr. RNA was extracted to measure by RT-qPCR the expression of CXCL10 and RSAD2, normalized to the housekeeping gene GAPDH. (D) Same experiment as in (C) except that HEK293 cells stably expressing UK-NSP1 were used. (E) HEK293 cells were transfected with indicated siRNA, infected with RRV or Wa (MOI = 1, 3 dpi), and harvested for RT-qPCR analysis examining the expression of viral gene NSP5, normalized to GAPDH. (F) Supernatants from (E) were collected at indicated time points and titrated by standard plaque forming unit assay. In all figures, experiments were repeated at least three times. Data are represented as mean ± SEM. Statistical significance is determined by Student’s t test (*p≤0.05; **p≤0.01; ***p≤0.001).

In the canonical NF-κB pathway, RelA (p65) and NF-κB1 (p50) subunits are held inactive and cytoplasmically sequestered by IκB. Following stimulation by pro-inflammatory cytokines or virus infection, SCF^β-TrCP^ complex targets IκB for degradation, releasing the brake on the p65/p50 heterodimer, whose translocation into the nucleus drives the expression of IFN and chemokines [7,8]. We hypothesized that Cul3 siRNA silencing, which abrogates NSP1-mediated β-TrCP degradation, should restore the NF-κB signaling. In line with our previous findings, at steady state, induced Wa-NSP1 led to a marked decrease in β-TrCP levels (Fig 3A, left). Cul1 and Rbx1 siRNA modestly rescued β-TrCP and Cul3 siRNA had the greatest effect (Fig 3A, left). Post TNF-α stimulation, β-TrCP promoted the degradation of IκBα, the major isoform of the IκB, but was counteracted by NSP1 expression (Fig 3A, right). Importantly, concurrent with Cul3 depletion, NSP1 was no longer able to induce β-TrCP degradation and IκBα degradation was restored (Fig 3A, right).

To determine whether restoring IκBα degradation by Cul3 inhibition would activate NF-κB, we next examined p65 nuclear translocation by immunofluorescence. In contrast to mock-treated cells, where we observed a significant amount of p65 nuclear staining in response to TNF-α or IL-1β treatment, p65 remained completely cytoplasmic in Wa-NSP1 expressing cells (Fig 3B, left). Rbx1 is the key E3 ubiquitin ligase of the SCF^β-TrCP^ complex that mediates IκBα degradation and its knockdown also retained p65 in the cytoplasm similar to Wa-NSP1 (Fig 3B, middle). Notably, Cul3 silencing up-regulated β-TrCP levels, resulting in IκBα degradation, and partially restored p65 translocation into the nucleus (Fig 3B, right). We further measured downstream NF-κB target genes, whose expression was strongly suppressed by RV NSP1. Wa-NSP1 significantly inhibited TNF-α-induced CXCL10 by down-regulation of β-TrCP without affecting its mRNA level (Fig 3C, left and S3C Fig). Concomitant with efficient Cul3 depletion (Fig 2E and S3A Fig), β-TrCP was no longer degraded and CXCL10 expression was restored to almost unperturbed levels (Fig 3C, left). Similarly, poly (I:C)-induced chemokine was also blocked by Wa-NSP1 and rescued by Cul3 knockdown (S3D Fig). The effect of Cul3 on the NF-κB pathway was specific since RSAD2, an interferon-stimulated gene (ISG), was not affected with IFN-β stimulation (Fig 3C, right). Besides Wa-NSP1, CXCL10 inhibition by UK-NSP1 was also lifted with Cul3 knockdown while having minimal effect on ISG expression (Fig 3D). Thus, our results demonstrate that reduced Cul3 levels abolished β-TrCP degradation, which in turn led to the restoration of IκB degradation, re-introduction of p65 nuclear translocation, and induction of chemokine expression.

Given the pivotal role of Cul3 in β-TrCP turnover, we further tested how its depletion and the resultant restoration of NF-κB signaling would affect RV growth. We measured both cell-associated viral RNA and extracellular virus titers for simian RRV and human Wa strains. Consistent with our prior findings, blocking Cul3 did not exert an inhibitory effect on RRV replication (Fig 3E and 3F), since the RRV NSP1’s ability to induce IRF3 degradation was Cul3-independent (Fig 2D). In marked contrast, both intracellular RNA genome copies and virus yield of Wa strain in the supernatant were negatively impacted by Cul3-CRL inhibition (Fig 3E and 3F), highlighting the importance of β-TrCP degradation in promoting human RV replication and the virus dependence on host Cul3-CRL complex.

### NSP1 co-localizes with Cul3 at the Golgi apparatus during virus infection

**Fig 4:**
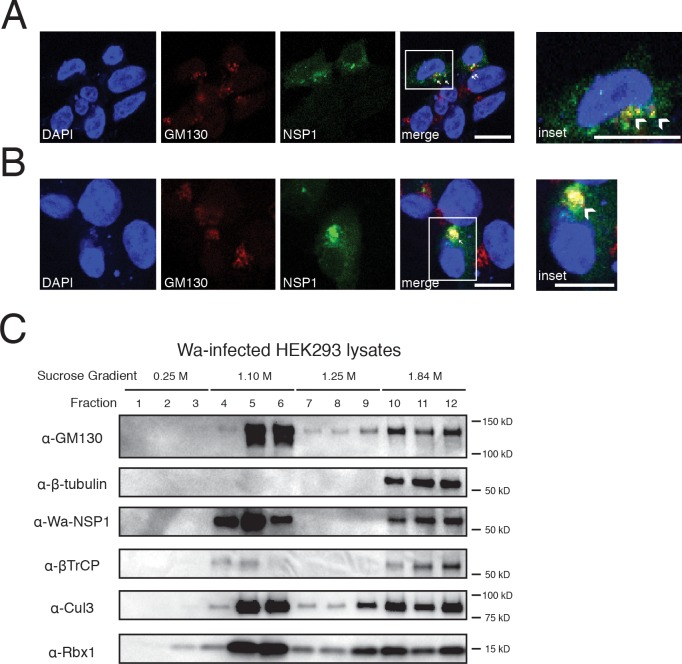
NSP1 co-localizes with CRL components at the Golgi apparatus. (A) HEK293 cells were transfected with pG-LAP6-Wa-NSP1 (green), and analyzed by confocal microscopy for the localization of the Golgi apparatus (GM130, red) and nucleus (DAPI, blue). Single cell zoom-in is shown in the inset. Co-localization (yellow) is highlighted by white arrowheads. Panels are single z slices with a scale bar of 15 μm. (B) Same experiment as in (A) except that cells were super-infected with Wa (MOI = 3, 12 hpi) prior to fixation. Single cell zoom-in is shown in the inset. Co-localization (yellow) is highlighted by white arrowheads. Panels are single z slices with a scale bar of 15 μm. (C) Lysates of Wa-infected HEK293 cells (MOI = 3, 24 hpi) were fractionated over a sucrose gradient using ultracentrifugation. Fractions were analyzed by western blot for the indicated antibodies. In all figures, experiments were repeated at least three times.

Based on the knowledge that NSP1 might be repurposing the host Cul3-CRL complex to induce β-TrCP degradation, we next sought to delineate the underlying molecular mechanisms. Multiple host and pathogen proteins contain the orthodox COPII sorting motif, composed of a transmembrane (TM) domain, a tyrosine residue and a spaced diacidic signal (S4A Fig). We noted two such motifs present in Wa-NSP1, one within the N-terminal RING-finger domain and the other at the very C-terminus. A further examination of NSP1 sequences revealed evolutionary conservation of these motifs (S4A Fig). The COPII coated vesicles are responsible for sorting and trafficking cargo out of the ER and into the Golgi apparatus [44]. Thus, we asked whether NSP1 localizes to the Golgi and whether this localization is necessary for its proper function. Since we did not have a good antibody that recognizes NSP1 by staining, we examined the localization of GFP-tagged NSP1. Indeed, supporting our hypothesis, we observed at least two populations of Wa-NSP1 with distinct subcellular localizations; one subset with bright punctuate clusters co-localized with the Golgi marker GM130 and the other subset characterized by a more diffuse and weaker signal (Fig 4A). In addition to GM130, we also observed clear co-localization of NSP1 with two alternative Golgi markers, golgin-97 and giantin (S4B Fig). We also examined NSP1 signal with Wa RV super-infection and were able to detect a similar Golgi localization pattern (Fig 4B). Despite the bright NSP1 clusters, the general Golgi morphology remained unchanged during transfection and early virus infection (Fig 4A and 4B). Additional staining further confirmed that NSP1 was not present at mitochondria (S4C Fig).

While Rbx1 is present in both nucleus and cytoplasm [45], Cul3 is known to be Golgi-resident [39,46]. To complement the immunofluorescence analysis, we performed biochemical isolation of subcellular organelles based on their sedimentation coefficient, which also revealed that during Wa RV infection, NSP1 co-fractionated with GM130 and β-tubulin, indicative of Golgi and cytoskeleton respectively (Fig 4C). Notably, in fractions 5 and 6, with the strongest GM130 signal, there was a substantial amount of Wa-NSP1, Cul3, Rbx1, and β-TrCP (Fig 4C). Interestingly, NSP1s from RRV and UK strain, both with COPII sorting motifs, also localized to the Golgi with a similar speck-like pattern (S4D Fig). Despite RRV-NSP1 localization to the Golgi and interaction with Cul3, it does not induce β-TrCP degradation, suggesting that Golgi localization and Cul3 interaction are insufficient for β-TrCP degradation.

### NSP1 serves a scaffold protein to connect Cul3 and β-TrCP

**Fig 5:**
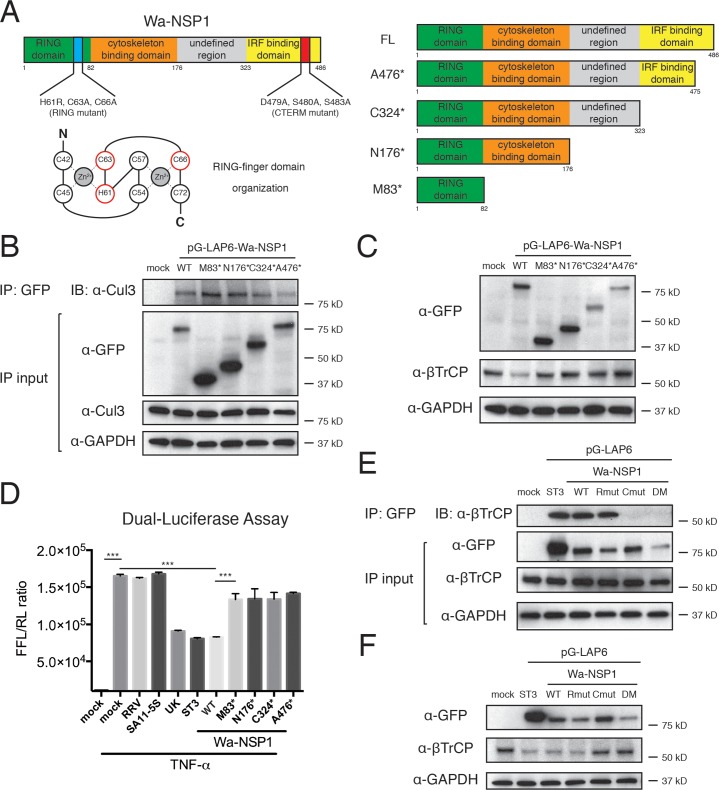
Wa-NSP1 interacts with host proteins Cul3 and β-TrCP. (A) Schematic of structural and functional domains within WT Wa-NSP1 and illustration of Wa-NSP1 point mutations and deletion mutants. (B) Lysates of HEK293 cells transfected with WT and mutant Wa-NSP1s were subject to immunoprecipitation using α-GFP antibody and probed with α-Cul3 antibody. (C) Lysates of HEK293 cells transfected with WT and mutant Wa-NSP1s were analyzed by western blot using indicated antibodies. (D) HEK293 cells were co-transfected with PRDII-luc, pRL-TK and Wa-NSP1 mutants, stimulated with TNF-α (10 ng/ml) for 6 hr, and harvested for Dual-Glo luciferase assay. Arbitrary units were determined by the ratio of firefly luciferase (FFL) to renilla luciferase (RL). (E) Lysates of HEK293 cells transfected with indicated pG-LAP6 plasmids and treated with 10 μM of lactacystin for 6 hr were subject to immunoprecipitation using α-GFP antibody and probed with α-β-TrCP antibody (Rmut: RING mutant, H61RC63AC66A; Cmut: CTERM mutant, D479AS480AS483A; DM: Double-Mutant, Rmut+Cmut). (F) Lysates of HEK293 cells transfected with indicated pG-LAP6 plasmids were analyzed by western blot using indicated antibodies. In all figures, experiments were repeated at least three times. Data are represented as mean ± SEM. Statistical significance is determined by Student’s t test (*p≤0.05; **p≤0.01; ***p≤0.001).

To directly interrogate how the interactions of NSP1, Cul3, and β-TrCP take place at the Golgi and what role NSP1 plays in this multi-protein complex, we designed a series of NSP1 mutants based on previous knowledge of NSP1 domains (Fig 5A). We examined their ability to: 1) localize to the Golgi; 2) interact with Cul3; and 3) cause β-TrCP degradation. Interestingly, M83* NSP1, with the minimal RING-finger domain, displayed Golgi localization but did not overlap with the actin filament staining, in contrast to WT NSP1 accumulation on the cytoskeleton [47]. (S5A and S5B Fig). The other mutants, N176*, C324* and A476*, which compared to M83* contains an additional cyto-binding domain, localized to Golgi and actin filaments similar to the WT protein (S5A Fig). Not only did all the Wa-NSP1 deletion mutants retain their Golgi localization (S5A Fig), they were also co-purified with endogenous Cul3 (Fig 5B), suggesting that within NSP1, the Cul3 binding domain also maps to the RING-finger domain. Strikingly, despite the unperturbed interaction with Cul3 and Golgi localization, none of these mutants were able to induce β-TrCP degradation as efficiently as the full-length Wa-NSP1 (Fig 5C), reminiscent of the NSP1 derived from simian RRV strain (Fig 1D and S4D Fig). Consistently, all the NSP1 deletion mutants failed to dampen the luciferase expression driven by the NF-κB signaling (Fig 5D). To further examine how the Golgi localization of NSP1 might affect its degradative activity, we generated an NSP1 mutant with the N-terminal RING-finger domain removed. This mutant, which we named RINGless NSP1, exhibited cytoplasmic staining and did not co-localize with the Golgi as did the WT protein (S6A Fig). Importantly, RINGless NSP1 also completely lost its ability to mediate β-TrCP degradation and failed to inhibit TNF-α induced NF-κB activation (S6B and S6C Fig). Altogether, these findings support our model that the Golgi localization and subsequent interaction with Cul3-CRL are necessary but not sufficient for β-TrCP degradation.

The only difference between WT and A476* NSP1 lies in the last 11 amino acids (Fig 5A), encompassing the reported PDL motif [21]. To pinpoint the precise residues underlying β-TrCP recognition, we introduced point mutations, producing a RING-finger domain mutant (Rmut) that disrupts both Zn^2+^ binding sites (Fig 5A), a C-terminal domain mutant (Cmut) that destroys the degron, and a double-mutant defective in both regions. Indeed, Cmut was unable to induce β-TrCP degradation due to loss of ability to bind to the substrate (Fig 5E and 5F), confirming the importance of degron in correctly locating β-TrCP [21]. This offers an explanation for why RRV-NSP1 and Cul3 deletion mutants had the capacity to localize to the Golgi, interact with Cul3, but were unable to mediate β-TrCP degradation. We believe that for RV NSP1 protein, Cul3 interaction and substrate recognition are executed by its N-terminal and C-terminal domains respectively. Therefore, these events do not necessarily have to be coupled together, accounting for the fact that certain strains (i.e. RRV) localize to the Golgi and interact with Cul3 but do not induce β-TrCP degradation. However, in contrast to the reported RING mutant C42A [21], Rmut that completely destroys the RING domain catalytic sites still retained the ability to degrade β-TrCP (Fig 5F). These findings, together with strong Rbx1 binding, are inconsistent with the hypothesis that NSP1 is the viral E3 ligase. Rather, our results suggest that NSP1 interacts with Cul3 and β-TrCP using its N- and C-terminal domains respectively and it functions as an adaptor protein to mediate the interaction between the two at the Golgi.

### Cul3-CRL mediates proteasomal degradation of NSP1 and β-TrCP

**Fig 6:**
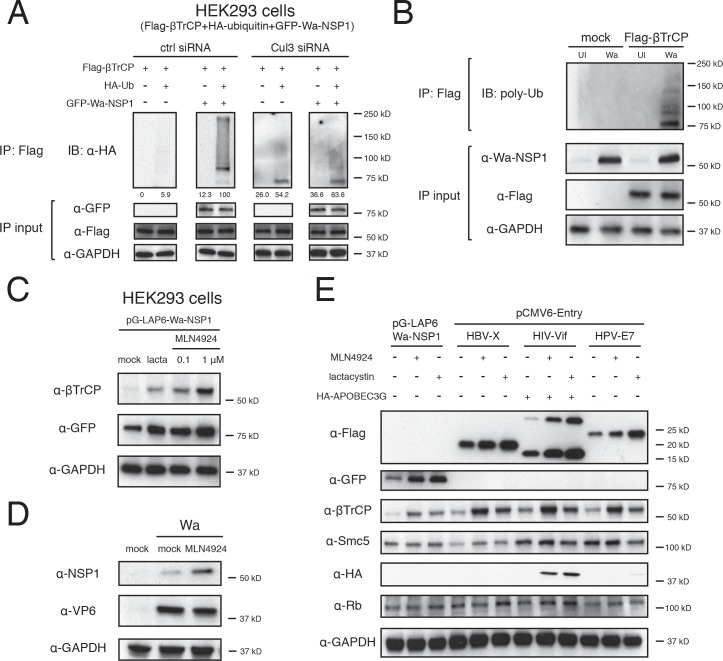
NSP1 and β-TrCP are targeted for proteasomal co-degradation. (A) HEK293 cells were transfected with control or Cul3 siRNA, and co-transfected with Flag-β-TrCP, HA-tagged Ubiquitin (Ub) and GFP-Wa-NSP1. Lysates were subject to immunoprecipitation using α-Flag antibody and probed with α-HA antibody. Top panel blots were quantified and the ubiquitination intensities were expressed as a percentage of the Ub level in the Ub+NSP1+ctrl siRNA lane, which is set to 100. (B) HEK293 cells were transfected with Flag-β-TrCP and infected with human RV Wa (MOI = 3, 24 hpi). Lysates were subject to immunoprecipitation using α-Flag antibody and probed with α-poly-Ub antibody (UI: uninfected). (C) HEK293 cells were transfected with pG-LAP6-Wa-NSP1, treated with lactacystin (10 μM) or MLN4924 (0.1 or 1 μM) for 24 hr, and harvested for western blot using indicated antibodies. (D) HEK293 cells were mock or Wa infected (MOI = 3, 12 hpi), treated with MLN4924 (1 μM) for 11 hr, and harvested for western blot using indicated antibodies. (E) HEK293 cells were co-transfected with HA-tagged APOBEC3G, pG-LAP6-Wa-NSP1 or pCMV6-Entry vector encoding Flag-tagged indicated viral proteins, treated with lactacystin (10 μM) or MLN4924 (1 μM) for 24 hr, and harvested for western blot using indicated antibodies. In all figures, experiments were repeated at least three times.

To better understand how the hijacked Cul3-CRL complex promotes β-TrCP turnover, we examined possible ubiquitin (Ub) modifications on β-TrCP. Only in the presence of Wa-NSP1 did we observe strong poly-Ub ladder pattern, from approximately 62 kD of the unconjugated protein all the way to the top of the gel (Fig 6A). Importantly, the Ub pattern was potently inhibited with Cul3 silencing, indicating an important role of the CRL in marking β-TrCP for ubiquitination (Fig 6A). We further confirmed this finding in the context of RV infection and β-TrCP was only Ub-modified during Wa infection, where NSP1 was expressed (Fig 6B).

Based on Ub modification and reduced β-TrCP levels, we speculated that β-TrCP might be shuttled to the proteasome for destruction. Indeed, none of the lysosome inhibitors blocked NSP1-mediated β-TrCP degradation (S7A Fig). On the other hand, although MG-132 was minimally effective (S7B Fig), lactacystin potently up-regulated β-TrCP levels (S7C and S7D Fig). Lactacystin, unlike MG132 and bortezomib, inhibits the proteasome through non-reversible covalent bonds at the N-terminus threonine residue in the β-1 subunit of the 20S proteasome [48], and this distinct mechanism of action could account for its efficacy. Carfilzomib, another irreversible inhibitor, was also effective at restoring β-TrCP levels (S7D Fig). Taken together, these experiments demonstrate that NSP1 utilizes Cul3-CRL complex to mark β-TrCP for degradation through the ubiquitin-proteasome pathway.

Surprisingly, concomitant with enhanced β-TrCP levels with proteasome or CRL inhibition, we observed a dose-dependent increase in Wa-NSP1 levels as well (Fig 6C). This is consistent with the previously observed up-regulation of Wa and ST3 NSP1 levels with Cul3 CRISPR-mediated partial depletion (S2E Fig, right panel), implying that besides β-TrCP, NSP1 itself was also regulated by the hijacked Cul3-CRL complex. We confirmed, in the context of wild type RV infection, that the stabilization of NSP1 by MLN4924 treatment was specific since the levels of Wa VP6, an RV structural protein, were not affected under these conditions (Fig 6D). We then postulated that other viral proteins that hijack host CRL complexes could also be employing the same mechanism for “co-destruction” with their substrates. To test this hypothesis, we examined the levels of HBV X protein (HBx), HIV accessory protein Vif, and HPV oncoprotein E7, which hijack Cul4 Cul5, and Cul2-CRL to respectively target Smc5/6 complex, APOBEC3G, and retinoblastoma tumor suppressor protein (Rb) for degradation [34,49,50]. Strikingly, treatment with either MLN4924 or lactacystin led to a marked increase in the levels of all three of these viral proteins and their corresponding substrates (Fig 6E). This is consistent with the previous report of poly-ubiquitination of HIV-Vif [51] and that all of these viral proteins invariably have a relative short half-life time: 30 min for HBx [52], 46 min for HIV-Vif [53], 55 min for HPV-E7 [54] and ~90 min for RV NSP1 [55]. Taken together, this data suggests a novel yet seemingly common strategy for viral non-structural proteins that utilize the host CRL complexes to attack selected host proteins to also “sacrifice” themselves in the process by undertaking a suicide mission that results in the co-degradation of the specific viral protein and its substrate, which in the case of RV are NSP1 and β-TrCP.

Previous studies indicate that Cul3 requires substrate recognition modules known as BTB-box proteins to constitute the BTB-Cul3-Rbx1 CRL complex [56]. To test whether BTB-box proteins are needed for NSP1 function, we transfected Wa-NSP1 cells with Cul3 mutants and examined β-TrCP degradation. Two of these mutants, ΔN41 and H2M/H5M, with the N-terminal 41 residues and helices 2, 5 removed respectively, are unable to make contact with BTB-box proteins [57]. We hypothesized that if NSP1 acts as the substrate recognition subunit of the multi-protein complex, there would be no necessity for BTB-box proteins. Indeed, ectopic expression of either ΔN41 or H2M/H5M Cul3 did not abolish β-TrCP degradation by NSP1 (S7E Fig), suggesting that in this scenario, Cul3 functions differently from the traditional CRL complex. However, expression of another dominant-negative mutant Cul3 ΔC (Cul3N418), which is defective in Rbx1 binding [58] efficiently restored β-TrCP levels in Wa-NSP1 expressing cells (S7E Fig), indicating that Rbx1 in association with Cul3 rather than Cul1-interacting Rbx1 primarily contributed to the E3 ligase activity.

### Inhibition of Cullin-E3 ligase activity blocks NSP1-induced β-TrCP degradation in human intestinal enteroids

**Fig 7:**
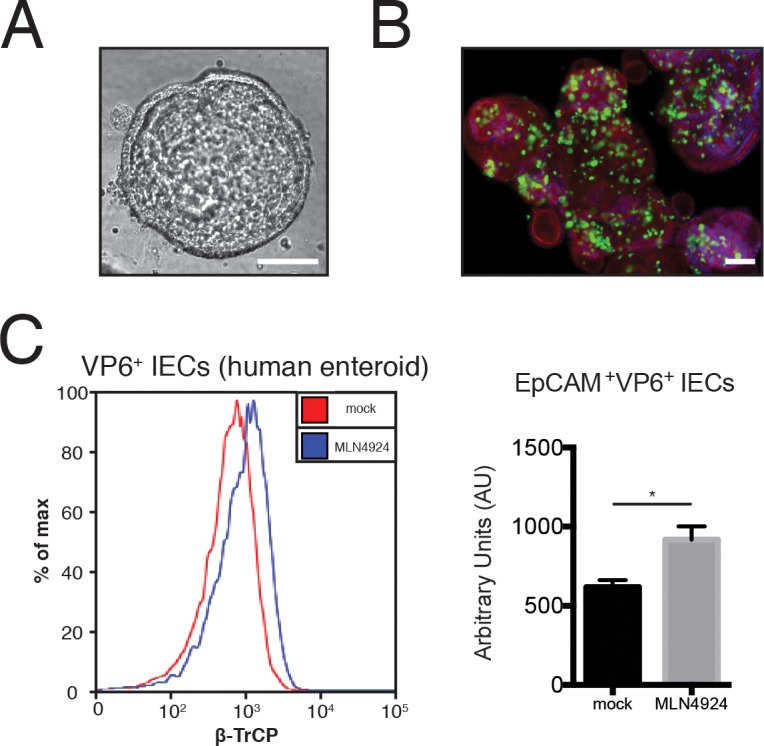
CRL inhibition reduces β-TrCP degradation by RV Wa infection in human intestinal enteroids. (A) Brightfield images of human enteroids cultured in 3D Matrigel matrix. Scale bar, 100 μm. (B) Enteroids were infected with human RV Wa strain (MOI = 3) for 16 hr and analyzed by confocal microscopy for viral antigen VP6 (green), cytoskeleton (phalloidin staining, red), and nucleus (DAPI, blue). Scale bar, 100 μm. (C) Enteroids were infected with human RV Wa strain (MOI = 1, 24 hpi) with or without MLN4924 treatment (1 μM, 24 hr). Trypsin-digested single cells were stained with viral antigen VP6, epithelium marker EpCAM and β-TrCP, and analyzed by flow cytometry. Fluorescent intensity of β-TrCP in EpCAM, VP6 double positive cells was quantified in FlowJo v8.8. In all figures, experiments were repeated at least three times. Data are represented as mean ± SEM. Statistical significance is determined by Student’s t test (*p≤0.05; **p≤0.01; ***p≤0.001).

Since most of our experiments were conducted in cell culture, we next assessed whether or not inhibition of Cul3 CRL activity could also rescue β-TrCP from NSP1 degradation in a more physiologically relevant system. We took advantage of nontransformed, three-dimensional human intestinal epithelial cell (IEC) organoids, which are derived from subject biopsies, consist entirely of IECs, and can recapitulate the biological architecture of the small intestine epithelium (Fig 7A) [59]. The human IEC enteroids fully supported vigorous replication and propagation of human RVs (Fig 7B). Importantly, consistent with our previous findings, MLN4924 treatment significantly up-regulated the levels of β-TrCP in human RV Wa-infected EpCAM^+^ IECs (Fig 7C), further corroborating the positive roles of host CRL in β-TrCP degradation by human RVs.

## Discussion

Rotavirus infection remains a global threat to public health, especially in underdeveloped countries where prophylactic vaccination has been only partially successful. This is due to many reasons including the inadequate understanding of several critical aspects of virus-host interactions. Although RV encodes only 11~12 proteins depending on the strain, assigning specific functions to individual RV proteins has been difficult in the absence of a tractable reverse-genetics system. In the present study, using an unbiased tandem AP-MS proteomics approach, we comprehensively investigate the host protein interaction network for six NSP1s (two human and four animal RV strains), the viral protein central to the antagonism of innate immune responses and contributing to host range restriction *in vivo*. This work provides mechanistic insights into how RVs intervene with the innate immune response and specifically how certain NSP1s effectively block NF-κB signaling by facilitating β-TrCP degradation.

**Fig 8:**
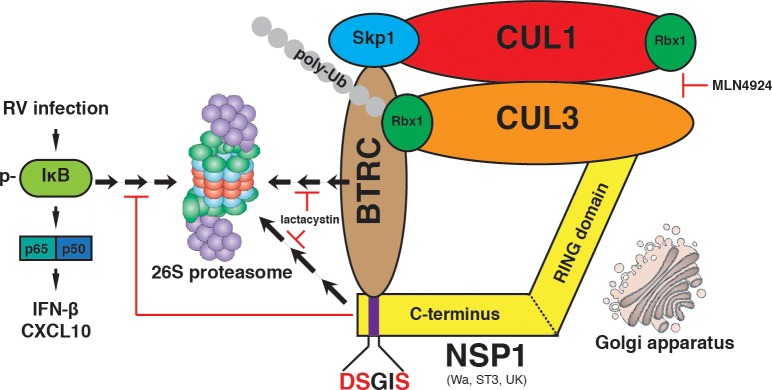
Model for NSP1 hijacking of Cul3-CRL to target β-TrCP for degradation. During RV infection, NSP1 co-localizes with Cul3 and Rbx1 at the Golgi apparatus, interacts with Cul3 and β-TrCP using its N- and C-terminal domains respectively, and promotes the co-degradation of β-TrCP and NSP1 at the proteasome. Blocking Cul3 (MLN4924) or proteasome (lactacystin) prevented β-TrCP turnover by RV NSP1. Cul3 inhibition leads to the restoration of IκB levels and NF-κB-driven gene expression.

Our work demonstrates, for the first time, that NSP1s from multiple RV strains strongly interact with the host CRL complex (Fig 1D) and that this interaction appears to take place at the Golgi (Fig 4). Co-localization of NSP1 and Golgi was demonstrated by immunofluorescence staining and gradient density co-sedimentation (Fig 4). In addition, deletion of a Golgi-localization signal containing region at the N-terminus of NSP1 eliminated Golgi localization and NSP1’s ability to degrade β-TrCP (S6 Fig). Additionally, our work demonstrates blocking Cul3 function, either by siRNA, inhibitor or a dominant-negative mutant, reduces or eliminates NSP1’s ability to cause β-TrCP degradation (Fig 2) and negatively impacts RV replication (Fig 3E). Along with Cul3 depletion, we observe a profound decrease in β-TrCP poly-ubiquitination (Fig 6A) and an increase in endogenous β-TrCP levels, both in cell culture and during actual RV infection in human IEC organoids (Fig 7C). Therefore, we have uncovered a novel mechanism that RV NSP1s utilize to degrade β-TrCP by rewiring the host Cul3-CRL complex. Although several viral proteins that interact with Cul3 have been previously reported [60–62], RV is unique in that NSP1 acts upstream in the NF-κB cascades by directly mediating the degradation of β-TrCP and itself (Fig 8). Interestingly, this co-destruction seems to be a rather common mechanism utilized by multiple viral proteins (Fig 6E). Vif, HBx, E7 and NSP1 are all, without exception, non-structural viral proteins, which might be more “expendable” after they have carried out their specific mission.

Our current study is particularly important because it resolves a long-standing controversy in the rotavirus field as to whether or not NSP1 is a viral E3 ubiquitin ligase. Despite the presence of a RING-finger domain, previous examination of its putative E3 ligase activity by the “gold standard” *in vitro* ubiquitination assay had not been successful. This could be due to 1) lack of proper E2 ubiquitin-conjugating enzyme(s), many of which are revealed in this proteome study (S1C Fig), 2) lack of additional co-factors, or 3) the possibility that NSP1 is not an E3 ligase by itself and its function demands the assistance from one or multiple host E3 ligase(s). Here, we present compelling experimental evidence that β-TrCP degradation is strictly dependent on Rbx1 (Fig 2E). Moreover, NSP1s with the catalytic sites mutated are nonetheless capable of mediating β-TrCP degradation (Fig 5F). Therefore, it appears that Wa-NSP1 does not function as an E3 ligase on its own but instead usurps the host CRL to degrade β-TrCP. It is noteworthy that RRV/ETD-NSP1s target IRF3 for degradation and this process is completely independent of Cul3 and Rbx1 (Fig 2D), highlighting a major difference of NSP1 dependence on host CRL during RV evolution and emphasizing the versatility of various RV strains in identifying substrates. It is interesting to propose whether the NSP1 protein, in the cases of RRV or ETD, might actually function as a *bona fide* E3 ligase. Future biochemical studies will be needed to clarify this issue.

Another interesting question is the rationale behind β-TrCP regulation by Cul3-CRL instead of its own Cul1-SCF complex. It is tempting to speculate the core E3 ligase Rbx1 in the SCF^β-TrCP^ complex might be spatially distant from β-TrCP (Fig 8). Possibly there exists steric hindrance for Cul1-asscoated Rbx1 to bend over and mark β-TrCP for poly-ubiquitination and the introduction of an “outsider” Cul3/Rbx1 helps to stabilize the complex and facilitate the addition of poly-Ub chains onto β-TrCP. Supporting this hypothesis, a Cul3 mutant that is unable to bind Rbx1 blocks β-TrCP degradation by NSP1 (S7E Fig). Cul1 is also required (Fig 2E) since its presence might stabilize the multi-protein complex. To completely resolve this issue will require follow-up work using atomic level Cryo-Electron Microscopy, which will give mechanistic insights into the precise interactions between these proteins. It would also be important to pinpoint the exact lysine residue(s) within β-TrCP and NSP1 that are modified for ubiquitination, and give guidance to generating β-TrCP mutants that are resistant to NSP1-mediated degradation.

During the revision of this manuscript, an independent study focusing on a similar topic was published by Lutz et al. [31]. A key finding of that study was that RV NSP1 interacts with host Cul1 and Cul3 in a strain-specific manner, which is completely consistent with our results. However, their second key conclusion was that Cul3 is not involved in the NSP1-mediated degradation of either IRF3 or β-TrCP. This finding is in clear contrast with our data. At this time, we cannot be certain as to the cause of the discrepancy. However, possible sources of the disparity in results might be due to the differences in methodology applied including: 1) doxycycline-inducible stable cell lines were used in our study as compared to a transient transfection approach in the Lutz paper; 2) we performed siRNA knockdown and inhibitor treatment in HEK293 cells as compared to MA104 cells in the Lutz paper; 3) NSP1 origins were not completely the same in the two studies (Wa, ST3 etc. here versus OSU etc. in the Lutz paper). Nevertheless, we reached our conclusion that the host Cul3-Rbx1 E3 ligase complex does play a critical role in mediating β-TrCP degradation by NSP1 on the basis of the following findings presented above: 1) multiple experimental tools were utilized including siRNA knockdown, CRISPR, chemical inhibitors and dominant-negative mutants; 2) we validated our results in multiple cell types including a primary human enteroid culture system; 3) we demonstrated that inhibition of Cul3 not only blocked β-TrCP degradation but also functionally restored the NF-κB pathway and inhibited Wa replication (Fig 3). Although we have not assigned a role for the other cullins that co-precipitated with NSP1, it is likely that they also assume important roles in different aspects of NSP1 biology.

In summary, we have applied tandem AP-MS to create a global network of RV NSP1-host PPIs, and this approach has provided a new and deeper understanding into how NSP1 efficiently antagonizes the NF-κB signaling by hijacking the host Cul3-CRL complex. By linking individual NSP1s to specific host processes, our work, in conjunction with the ongoing development of an RV reverse genetics, will set the stage to test the functional role of NSP1 both *in vitro* and *in vivo*. Finally, this high-resolution comparative proteomics methodology has broad applicability to the study of viral pathogenesis in general and has the potential to uncover, in an unbiased manner, druggable cellular pathways that are required for efficient replication by various viruses.

## Materials and Methods

### Cells and reagents

Human embryonic kidney HEK293 cells were obtained from American Type Culture Collection (ATCC) and cultured in DMEM supplemented with 10% FBS, 2 mM L-glutamine, 100 IU/ml of penicillin and 100 μg/ml of streptomycin. HEK293-NSP1 stable cell lines were generated from HEK293 cells and cultured in complete DMEM in the presence of puromycin (0.5 μg/ml). Expression of NSP1 was induced by doxycycline (1 μg/ml) treatment for 24 hr. African Green Monkey kidney MA104 cells were obtained from ATCC and cultured in Medium 199 supplemented with 10% FBS, 2 mM L-glutamine, 100 IU/ml of penicillin and 100 μg/ml of streptomycin. Cells were stimulated with TNF-α (10 ng/ml), IL-1β (10 ng/ml), IFN-β (100 U/ml), poly (I:C) LMW/LyoVec (100 ng/ml) for 15 min or 6 hr for either western blot/immunofluorescence or QPCR quantification, respectively. Cells were treated with either small-molecule proteasome inhibitors (10 μM) for 12 hr: MG-132, bortezomib, carfilzomib, VR23, celastrol, curcumin from Selleckchem, lactacystin and epoxomicin from Enzo Life Sciences; or lysosome inhibitors (10 μM) for 12 hr: chloroquine (InvivoGen), concanamycin A (Enzo), bafilomycin A (Sigma). NEDD8 activating enzyme inhibitor MLN4924 (Millipore) was reconstituted in DMSO (stock concentration: 20 mM) and used at the range of 100 nM to 10 μM.

### Tandem affinity purification and mass spectrometry

To best optimize the LAP purification procedure and minimize the possibility of carryover, we have standardized the growth of cells, the preparation of extracts, and the method of tandem affinity purification [27]. Briefly, stable LAP cell lines were harvested using detergent. Lysates were clarified at 43,000 rpm and subjected to anti-GFP immunoprecipitation. Bound proteins were eluted from antibody beads using TEV protease, recaptured on S-protein agarose (Novagen), and eluted in 2x NuPAGE sample buffer (Invitrogen). Following purification, great care is taken to ensure a lack of contamination from both environmental sources and from other purified proteins. Each purified set of interacting proteins is separated on an individual 4–12% Bis-Tris polyacrylamide gel and stained with Coomassie brilliant blue. 293 cells samples were run into gels for 20-40mm and divided into 20–40 x1 mm slices. Each excised lane was reduced, propionamidated and digested with trypsin. Peptide identification of each digestion mixture was performed by microcapillary reversed-phase HPLC nanoelectrospray tandem mass spectrometry (mLC-MS/MS) on an LTQ-Orbitrap Elite or Fusion mass spectrometer (ThermoFisher Scientific, Waltham, MA). The Orbitrap repetitively surveyed an m/z range from 395 to 1600, while data-dependent MS/MS spectra on the twenty (Velos) or ten (XL) most abundant ions in each survey scan were acquired in the linear ion trap. MS/MS spectra were acquired with relative collision energy of 30%, 2.5-Da isolation width, and recurring ions dynamically excluded for 60 s.

Peptide sequencing and protein inference was facilitated using Byonic following an initial quality control analysis using Preview (Protein Metrics, San Carlos, CA). In a typical Byonic analysis, a 12 ppm mass tolerance for precursor ions and 0.4 Da mass tolerance for fragment ions against a species specific (mouse or human).fasta file derived from the NCBI Genbank protein database with custom sequences added for specific tagged bait protein sequences. Fully specific tryptic peptides were accepted, with up to two missed cleavages, and allowing for various common modifications such as methionine oxidation and acetylation of protein N-termini, as well as modifications specific to the pathways investigated. Both protein- and peptide-level false discovery rates were held to an estimated false discovery rate (FDR) of <1% using a reverse decoy database strategy.

### TAP/MS data network generation

For individual genes identified in each AP/MS sample, we assigned a normalized spectral abundance factor (NSAF) to each gene (g) [63].

$$NSAF_{g}=\frac{P_{g}/L_{g}}{\sum_{i}P_{i}/L_{i}}$$

The S_g_ for a gene is the number of peptides P_g_ divided by the total number of peptides observed from other genes in the dataset. This includes all peptides except those known to derive from the bait protein and those derived from known exogenous proteins. We exclude, for example, proteins commonly found in human skin and those added during sample preparation. We then divide this score by the mean length of NCBI reference protein isoforms from *g (L*_*g*_ *)* in amino acids. Using a set of eight negative control datasets, we systematically search for genes whose score in an experimental data set is highly unlikely. These filtered genes included in the attached Cytoscape (http://www.cytoscape.org) network file, and a manually curated, simplified subset of these are shown in Fig 1.

### Virus infection

Human RV Wa strain and simian RV RRV strain were propagated in MA104 cells as previously described [64]. Viruses were activated by trypsin (5 μg/ml) at 37°C for 20 min prior to infection. Cells were washed with serum-free medium (SFM) twice and incubated with RV at different MOIs at 37°C for 1 hr. After removal of RV inoculum, cells were washed once with SFM, cultured in either complete medium or SFM and harvested at different time points for QPCR, western blot analysis or plaque assay.

### Plasmids

pcDNA3-HA-Rbx1 (#19897), pcDNA3-Myc-Cul3 (#19893), Cul3-H2M/H5M (#21591), Cul3-deltaN41 (#21590), pcDNA3-DN-hCUL3-Flag (#15820), Flag-β-TrCP (#10865), HA-tagged Ubiquitin (#17608) were obtained from Addgene. pcDNA3-HA-APOBEC3G was a kind gift from Dr. Reuben Harris (University of Minnesota). pCMV6-Entry encoding HIV-1-Vif (VC101719), HBV X protein (VC102194), HPV E7 protein (VC101903) were purchased from Origene. Point mutations and pre-mature stop codons in Wa-NSP1 were introduced into pENTR221 vector using QuikChange II Site-Directed Mutagenesis Kit (Agilent) and shuttled into pG-LAP6 destination vector using LR recombination reaction (Thermo Fisher).

### DNA and siRNA transfection

DNA transfection on HEK293 cells was performed using Lipofectamine 3000 reagent (Thermo) at the lipid: DNA ratio of 3:1 [65]. siRNA transfection was performed using RNAiMAX (Thermo) according to the reverse transfection protocol. Briefly, 1.2 μl of 5 μM siRNA was mixed with 1 μl RNAiMAX in 100 μl OptiMEM and incubated in 24-well plate at RT for 20min. 5×10^4^ HEK293 cells in 500 μl Ab-free DMEM were then added to each well. Further treatment was performed at least 48 hr post transfection. Different siRNA used in this study was listed in S7 Table.

### CRISPR-mediated Cul3 depletion

HEK293 cells were transiently transfected with pSpCas9(BB)-2A-GFP (PX458), encoding an sgRNA targeting the 7th coding exon of Cul3. Single GFP-positive cells were sorted to individual wells of six 96-well plates using a FACSAria II flow cytometer (BD) and genomic DNA extracted using the QuickExtract kit (Epicentre). The targeted genomic region was amplified by specific PCR primers (S7 Table) using Phusion Hot Start II DNA polymerase (NEB), cloned into Zero Blunt TOPO vector (Thermo). Six independent colonies were extracted with Miniprep (Qiagen) and subject to Sanger sequencing.

### RNA isolation and real-time quantitative PCR

Total RNA was harvested and extracted using RNeasy Mini Kit (Qiagen) as previously described [66]. In brief, RNA was converted to cDNA using High Capacity cDNA Reverse Transcription Kit (Applied Biosystems). QPCR was performed using the Stratagene Mx3005P (Agilent) with each reaction composed of cDNA reverse-transcribed from 50 ng of total RNA, 12.5 μl of Power SYBR Green master mix (Applied Biosystems), and 200 nM both forward and reverse primers in a total volume of 25 μl [67]. SYBR Green primers used in this paper are listed in S7 Table. The Taqman assay for determining viral gene NSP5 expression using a Brilliant III UltraFast QPCR Master Mix kit (Agilent Technologies) was performed as previously described [68].

### Luciferase reporter assay

HEK293 cells in 24-well plates (>90% confluency) were co-transfected with 1 μg of pG-LAP6 plasmids, 0.4 μg of PRDII-Luc (NF-κB-Luc), and 0.1 μg of pRL-TK. At 48 hr post transfection, cells were stimulated with TNF-α (10 ng/ml) for 6 hr and lysed in 100 μl Passive Lysis Buffer for dual-luciferase measurement following the manufacturer’s protocol (Promega).

### SDS-PAGE and western blot

SDS-PAGE was performed as previously described [69]. In brief, protein lysates were harvested in RIPA buffer, mixed with 2×Laemmli Buffer, boiled at 95°C for 5 min, and separated on pre-cast SDS-PAGE gels (Biorad). Silver Staining was performed using the Pierce Silver Stain Kit (#24612, Thermo) according to the manufacturer’s instructions. Western blot was conducted with wet-transfer onto nitrocellulose membranes, blocked with TBST with 5% FBS or 5% milk, and incubated with primary antibodies against β-TrCP (clone D13F10, Cell Signaling Technology, hereon abbreviated as CST); Cul1 (#4995, CST); Cul2 (A302-476A, Bethyl Lab); Cul3 (#2759, CST); Cul4A (#2699, CST); Cul4B (A303-863A, Bethyl Lab); Cul5 (A302-173A, Bethyl Lab); Flag (clone M2, Sigma); GAPDH (clone poly6314, Biolegend); GFP (#2555, CST); HA (clone C29F4, CST); HECTD1 (A302-908A, Bethyl Lab); IκBα (#9242, CST); IRF3 (clone D6I4C, CST); MAVS (A310-243A, Bethyl Lab); Myc (clone 71D10, CST); Nrf2 (clone D1Z9C, CST); Rb (clone 4H1, CST); Rbx1 (#4397, CST); Smc5 (A300-236A, Bethyl lab); TRAF2 (clone C192, CST); poly-Ub WT (clone P4D1, CST); rabbit polyclonal antibody against Wa-NSP1 was a kind gift from the Patton lab (University of Maryland); Secondary incubation was performed with anti-rabbit (#7074), or anti-mouse (#7076) IgG HRP-linked antibodies. Proteins were visualized using Clarity ECL substrate (#170–5061, Biorad), Amersham Hyperfilm (GE Healthcare) and STRUCTURIX X-ray film processor (GE Healthcare). The intensity of bands in western blot was quantified by densitometry using ImageJ.

### Immunoprecipitation

Cells were rinsed with ice-cold PBS and protein lysates were harvested in 1×lysis buffer (#9803, CST) supplemented with 1 mM PMSF. Lysates were first incubated with Pierce Protein A/G Magnetic beads (#88802, Thermo) at 4°C for 1 hr. Pre-cleared lysates were collected for IP input control and the rest were then incubated with Normal Rabbit IgG Polyclonal Antibody control (#12–370, Millipore) or primary antibodies against Cul3 (A301-109A, Bethyl Lab); Flag (M2 affinity gel, A2220, Sigma); GFP (ab290, Abcam); HA (clone C29F4, CST); Myc (clone 71D10, CST) at 4°C overnight. Antibody/lysates were further incubated with magnetic beads at RT for 30 min. The complex was washed with 1×lysis buffer for at least three times before added to elution buffer, which was prepared using 3×Blue Loading Buffer mixed with 30×DTT at 10:1 ratio (#7722, CST). Samples were boiled at 95°C for 5 min and supernatants were collected after centrifugation at 14,000 rpm at 4°C for 1 min. For western blot, mouse anti-rabbit Conformation Specific antibody (clone L27A9, CST) was used instead of traditional secondary antibodies.

### Immunofluorescence

2×10^4^ HEK293 cells were seeded into poly-D-lysine coated chamber slides (Nunc, Sigma) 2 days prior to experiments. Cells were cultured in the presence of MitoTracker Deep Red FM (M22426, Thermo) or LysoTracker Red DND-99 (L7528, Thermo) at 37°C for 30 min. RV-infected or plasmid transfected cells were rinsed with ice-cold PBS (with addition of Ca^2+^) and fixated in 4% PFA at RT for 10min. Cells were then washed with PBS and incubated with primary antibodies against with α-Flag Alexa-555 (#3768, CST); α-Myc Alexa-594 (#9483, CST); α-HA Alexa-647 (#3444, CST); GM130 (clone D6B1, CST); p65 (clone D14E12, CST) in IFA buffer (3% BSA, 1% saponin, 1% triton X-100, and 0.02% sodium azide in water) at RT for 1 hr. After washing with IFA buffer three times, secondary incubation was performed with chicken anti-rabbit-Alexa-594 (A21442, Thermo) at RT for 30 min protected from light. Cells were again washed with IFA buffer three times and PBS three times before staining with phalloidin Alexa-555 (#8953, CST) at RT for 15 min. Stained cells were washed with PBS, mounted with Antifade Mountant with DAPI (P36962, Thermo), and imaged with Zeiss LSM 710 Confocal Microscope. Micrographs were analyzed and co-localization co-efficient was determined by signal overlay based on the voxels and their intensities using Volocity software v5.3.2 (PerkinElmer).

### Human intestinal enteroid culture

Duodenal derived primary human intestinal enteroids were kindly provided by Dr. Calvin Kuo (Stanford University). The methods for enteroid culture and RV infection were similar to previous publication [70]. Briefly, 3D culture of intestinal enteroids in matrigel (Corning) was maintained in growth media made of advanced DMEM-F/12 media supplemented with several growth factors including epidermal growth factor, Noggin, R-spondin, Wnt3A, nicotinamide, gastrin I, SB202190, B27 supplement, N2 supplement and acetylcysteine. Two days prior to RV infection, enteroid was switched to differentiation media, which is growth media without Wnt3A, BS202190, nicotinamide and 50% reduction of Noggin and R-spondin. The enteroids were then lightly treated with TrypLE (Gibco) to remove matrigel and infected with human RV Wa strain (MOI = 1) for 1 hr at 37°C. After incubation, new matrigel was added to Wa-infected enteroids for 3D culture and the infected enteroids were cultured in differentiation media for a total of 24 hr at 37°C in 5% CO_2_ incubator. The enteroids were treated with TrypLE again to obtain single cell suspension before staining.

### Flow cytometry

Human intestinal enteroids were infected with Wa (MOI = 1) and treated with MLN4924 (1 μM) for 24 hr prior to harvest. Cells were stained with Live/Dead Aqua Kit (L34957, Thermo), and primary Ab against β-TrCP (clone 2H2, Novus Biologicals), secondary Ab goat-anti-mouse-APC (poly4053, Biolegend), and PE-conjugated anti-human CD326 (EpCAM) antibody (#324206, Biolegend). FITC-conjugated mouse monoclonal (1E11) antibody against VP6 was generated in our lab and previously characterized [14]. Fluorescence was measured with BD LSR II flow cytometer and data was analyzed with FlowJo Software v8.8.7 (TreeStar).

### Statistical analysis

The results were shown as means ± SEM. Statistical significance was determined by Student's t test using Prism 6 (GraphPad Software). Significant differences are indicated on figures (*p≤0.05; **p≤0.01; ***p≤0.001).

## Supporting Information

S1 Fig Proteomics analysis reveals interaction of E2 ubiquitin-conjugating enzymes with NSP1. Proteins that were presented in Fig 1C were listed here. Each lane in the SDS-PAGE gel was excised into 8 pieces from top to bottom and the number of strip corresponds to the size of the target protein. The coverage indicates the percentage of protein identified by tryptic peptides after mass spectrometry analysis. (B) Lysates of HEK293 cells co-transfected with indicated pG-LAP6-NSP1 plasmids and Flag-β-TrCP were analyzed by western blot using indicated antibodies. (C) Heat map summary of all the E2 proteins that bind to different NSP1s. The color corresponds to the number of peptides identified in the AP-MS experiments.
(TIF)

S2 Fig Rbx1 and Cul3 are not involved in RRV-NSP1 mediated IRF3 degradation but contribute to Wa-NSP1 induced β-TrCP degradation. (A) HEK293 cells stably expressing RRV-NSP1 were transfected with indicated siRNA, and treated with doxycycline at indicated concentrations for 24 hr. Western blot was performed to analyze the lysates using the indicated antibodies. (B) HEK293 cells stably expressing Wa-NSP1 were transfected with indicated siRNA, and treated with doxycycline. Western blot was performed to analyze the lysates using the indicated antibodies (FW11: FBXW11; HD1: HECTD1). (C) Same experiment as in (B) except that different siRNA was used. (D) MA104 cells were transfected with indicated siRNA, infected with RRV or Wa (MOI = 3, 12 hpi) and harvested for western blot analysis using indicated antibodies. Blots were quantified and the level of β-TrCP is normalized to the loading control GAPDH. The ratio in uninfected, control siRNA-transfected cells is set to 1. (E) Genotyping of CRISPR-induced incomplete Cul3 knockout HEK293 cells by Sanger sequencing showing the mutated locus (frameshifts) in multiple alleles and the wild-type reference (left panel). Wild-type HEK293 (WT) and HEK293 cells heterozygous (Het) for Cul3 were transfected with pG-LAP6-Wa-NSP1 or pG-LAP6-ST3-NSP1 and analyzed by western blot using indicated antibodies (right panel). In all figures, experiments were repeated at least three times.
(TIF)

S3 Fig Cul3 siRNA silencing restores chemokine expression via NF-κB signaling. (A) HEK293 cells were transfected with indicated siRNA, and total RNA was extracted to measure by RT-qPCR the expression of indicated host genes, normalized to the levels of GAPDH. (B) Same experiment as in (A), except that the protein levels of indicated host genes were measured by western blot using indicated antibodies (HD: HECTD1). (C) HEK293 cells stably expressing Wa-NSP1 were transfected with indicated siRNA, treated with doxycycline, and stimulated with poly (I:C) (100 ng/ml) for 6 hr. RNA was extracted to measure by RT-qPCR the expression of GFP and β-TrCP, normalized to the levels of GAPDH. (D) Same experiment as in (C), except that the expression of CCL5 and CXCL10 was measured by RT-qPCR and normalized to GAPDH. In all figures, experiments were repeated at least three times. Data are represented as mean ± SEM. Statistical significance is determined by Student’s t test (*p≤0.05; **p≤0.01; ***p≤0.001).
(TIF)

S4 Fig NSP1 localizes to the Golgi apparatus. (A) Illustration of amino acid sequences within exemplary proteins that direct their incorporation into COPII-coated vesicles. VSV-G, vesicular stomatitis virus glycoprotein; GLUT4, glucose transporter type 4; LDLR, low-density lipoprotein receptor; ASGPR-H1, asialoglycoprotein receptor 1. Tyrosine (or other resides at the same location) and diacidic signal are highlighted in cyan and green respectively. Phosphodegron-like motif is highlighted in red. (B) HEK293 cells were transfected with pG-LAP6-Wa-NSP1 (green), and stained with Golgi markers golgin-97 (red, top panel) or giantin (red, bottom panel), and nucleus (DAPI, blue). Co-localization (yellow) is highlighted by white arrowheads. Panels are single z slices with a scale bar of 15 μm. (C) HEK293 cells were transfected with pG-LAP6-Wa-NSP1 (green), and analyzed by confocal microscopy for mitochondria (mito, red) and nucleus (DAPI, blue). Panels are single z slices with a scale bar of 10 μm. (D) HEK293 cells were transfected with pG-LAP6-RRV-NSP1 (green, top panel) or pG-LAP6-UK-NSP1 (green, bottom panel), and analyzed by confocal microscopy for the localization of Golgi (GM130, red), cytoskeleton (cyto, grey), and nucleus (DAPI, blue). Co-localization (yellow) is highlighted by white arrowheads. Panels are single z slices with a scale bar of 10 μm. In all figures, experiments were repeated at least three times.
(TIF)

S5 Fig RING-finger domain of NSP1 mediates Golgi localization. (A) HEK293 cells were transfected with pG-LAP6-Wa-NSP1 mutants M83*, L176*, C324*, and A476* (green), and analyzed by confocal microscopy for the localization of Golgi (GM130, red), cytoskeleton (cyto, grey), and nucleus (DAPI, blue). Co-localization (yellow) is highlighted by white arrowheads. Panels are single z slices with a scale bar of 10 μm. (B) The co-efficient value of co-localization of Wa-NSP1 protein (WT and mutants) and different cellular organelles was calculated in Volocity v5.2 on the basis of at least 20 micrographs. Co-localization of cytoskeleton and nucleus is set to be 20 (dotted line) and serves as the negative control. In all figures, experiments were repeated at least three times. Data are represented as mean ± SEM. Statistical significance is determined by Student’s t test (*p≤0.05; **p≤0.01; ***p≤0.001).
(TIF)

S6 Fig NSP1 lacking its RING-finger domain does not localize to Golgi nor degrade β-TrCP. (A) HEK293 cells were transfected with pG-LAP6-Wa-NSP1 WT or mutant without RING-finger domain (RINGless, green), and analyzed by confocal microscopy for the localization of Golgi (GM130, red), and nucleus (DAPI, blue). Co-localization (yellow) is highlighted by white arrowheads. Panels are single z slices with a scale bar of 10 μm. (B) Lysates of HEK293 cells transfected with WT and RINGless Wa-NSP1s were analyzed by western blot using indicated antibodies. (C) HEK293 cells were co-transfected with PRDII-luc, pRL-TK and Wa-NSP1 mutants, stimulated with TNF-α (10 ng/ml) for 6 hr, and harvested for Dual-Glo luciferase assay. Arbitrary units were determined by the ratio of firefly luciferase (FFL) to the transfection control renilla luciferase (RL). In all figures, experiments were repeated at least three times. Data are represented as mean ± SEM. Statistical significance is determined by Student’s t test (*p≤0.05; **p≤0.01; ***p≤0.001).
(TIF)

S7 Fig β-TrCP degradation by NSP1 is blocked by proteasome inhibitors. (A) Lysates of HEK293 cells stably expressing Wa-NSP1 were treated with doxycycline and indicated lysosome inhibitors, and analyzed by western blot using indicated antibodies. Blots were quantified and the level of β-TrCP is normalized to loading control GAPDH. The ratio of β-TrCP versus GAPDH in mock-treated cells is set to 1. (B) Wild-type HEK293 cells or HEK293 cells stably expressing Wa-NSP1 were transfected with indicated siRNA and treated with doxycycline and MG132. Lysates were harvested for western blot analysis using indicated antibodies. (C) Same experiment as in (A) except that proteasome and translation inhibitors were used (MG: MG132; bort: bortezomib; lact: lactacystin; epox: epoximicin; CHX: cycloheximide). (D) Same experiment as in (A) except that proteasome and E1 inhibitors were used (VR: VP23; carfil: carfilzomib; curc: curcumin; celas: celastrol; lact: lactacystin; PYR: PYR-41). (E) HEK293 cells stably expressing Wa-NSP1 were transfected with plasmids encoding WT or mutant Cul3, treated with doxycycline and harvested for western blot analysis using indicated antibodies. In all figures, experiments were repeated at least three times. Data are represented as mean ± SEM. Statistical significance is determined by Student’s t test (*p≤0.05; **p≤0.01; ***p≤0.001).
(TIF)

S1 Table List of host proteins identified by AP-MS that interact with RRV-NSP1. (XLSX)

S2 Table List of host proteins identified by AP-MS that interact with ETD-NSP1. (XLSX)

S3 Table List of host proteins identified by AP-MS that interact with UK-NSP1. (XLSX)

S4 Table List of host proteins identified by AP-MS that interact with WA-NSP1. (XLSX)

S5 Table List of host proteins identified by AP-MS that interact with ST3-NSP1. (XLSX)

S6 Table List of host proteins identified by AP-MS that interact with SA11-5S-NSP1. (XLSX)

S7 Table List of oligonucleotides used in this study. (DOCX)
