# Supplementary figures and images for: Comparative Proteomics Reveals Strain-Specific β-TrCP Degradation via Rotavirus NSP1 Hijacking a Host Cullin-3-Rbx1 Complex
Source: PLoS Pathog. 2016 Oct 5;12(10):e1005929. doi: 10.1371/journal.ppat.1005929 (PMC5051689; doi:10.1371/journal.ppat.1005929)

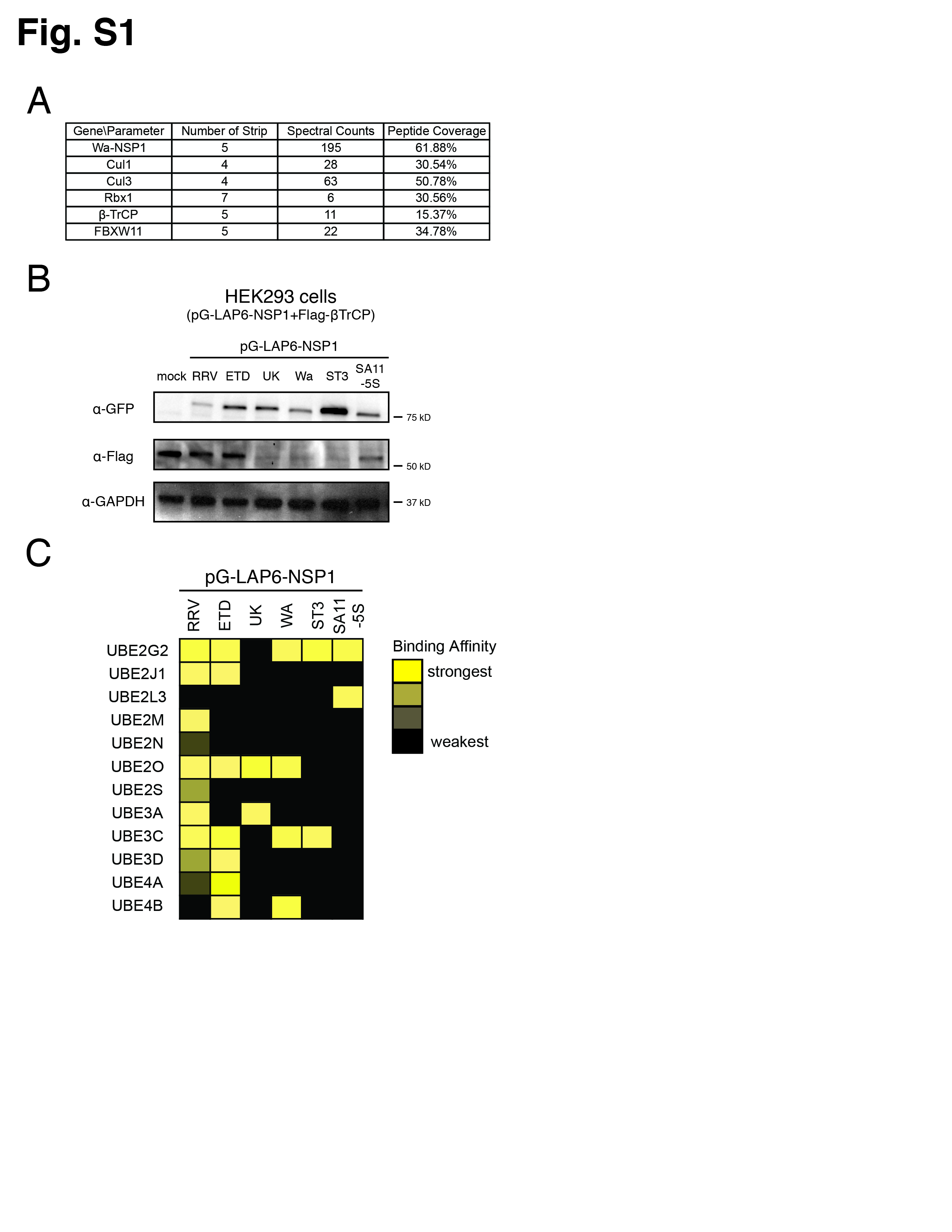

Supplement: S1 Fig — Proteins that were presented in Fig 1C were listed here. Each lane in the SDS-PAGE gel was excised into 8 pieces from top to bottom and the number of strip corresponds to the size of the target protein. The coverage indicates the percentage of protein identified by tryptic peptides after mass spectrometry analysis. (B) Lysates of HEK293 cells co-transfected with indicated pG-LAP6-NSP1 plasmids and Flag-β-TrCP were analyzed by western blot using indicated antibodies. (C) Heat map summary of all the E2 proteins that bind to different NSP1s. The color corresponds to the number of peptides identified in the AP-MS experiments. (TIF) [file ppat.1005929.s001.tif]

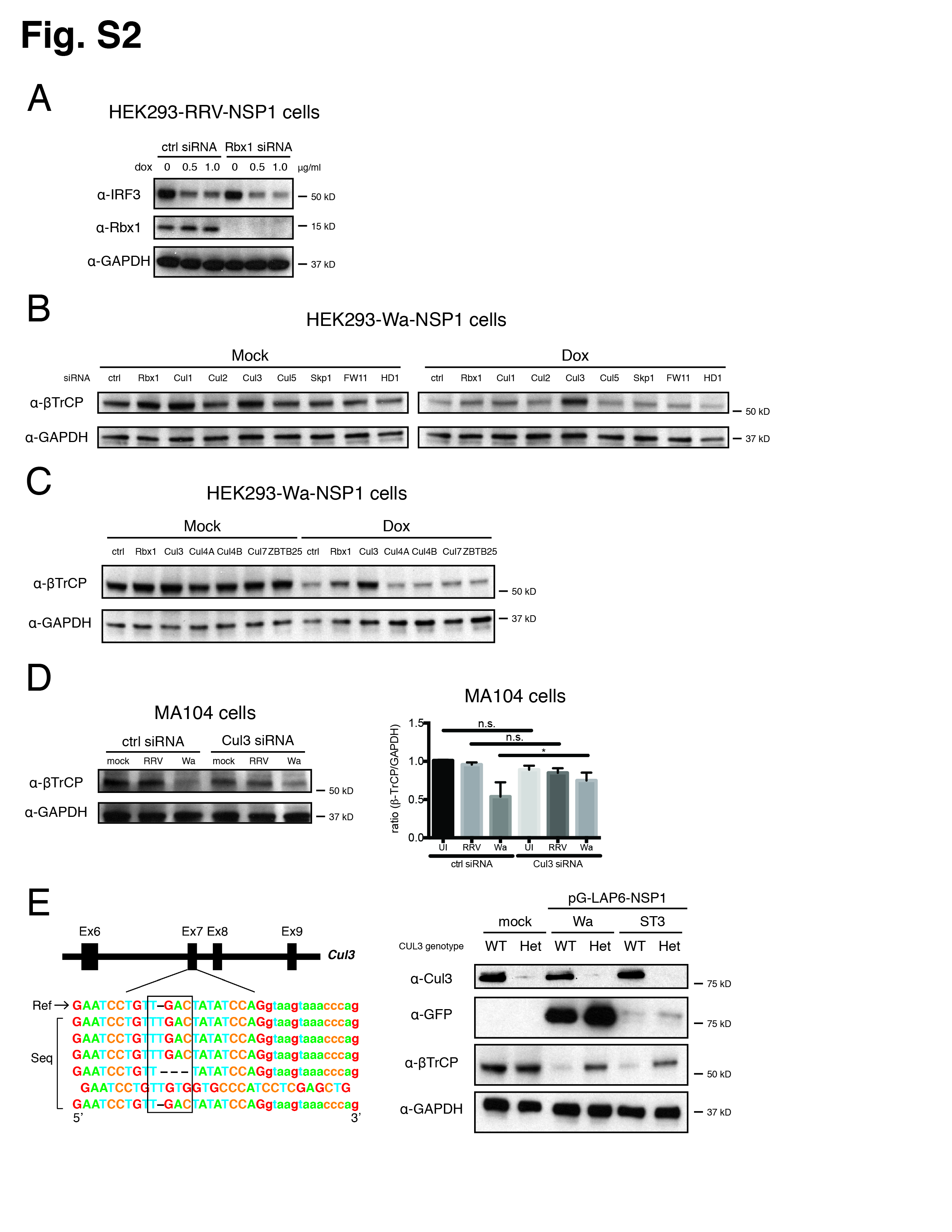

Supplement: S2 Fig — (A) HEK293 cells stably expressing RRV-NSP1 were transfected with indicated siRNA, and treated with doxycycline at indicated concentrations for 24 hr. Western blot was performed to analyze the lysates using the indicated antibodies. (B) HEK293 cells stably expressing Wa-NSP1 were transfected with indicated siRNA, and treated with doxycycline. Western blot was performed to analyze the lysates using the indicated antibodies (FW11: FBXW11; HD1: HECTD1). (C) Same experiment as in (B) except that different siRNA was used. (D) MA104 cells were transfected with indicated siRNA, infected with RRV or Wa (MOI = 3, 12 hpi) and harvested for western blot analysis using indicated antibodies. Blots were quantified and the level of β-TrCP is normalized to the loading control GAPDH. The ratio in uninfected, control siRNA-transfected cells is set to 1. (E) Genotyping of CRISPR-induced incomplete Cul3 knockout HEK293 cells by Sanger sequencing showing the mutated locus (frameshifts) in multiple alleles and the wild-type reference (left panel). Wild-type HEK293 (WT) and HEK293 cells heterozygous (Het) for Cul3 were transfected with pG-LAP6-Wa-NSP1 or pG-LAP6-ST3-NSP1 and analyzed by western blot using indicated antibodies (right panel). In all figures, experiments were repeated at least three times. (TIF) [file ppat.1005929.s002.tif]

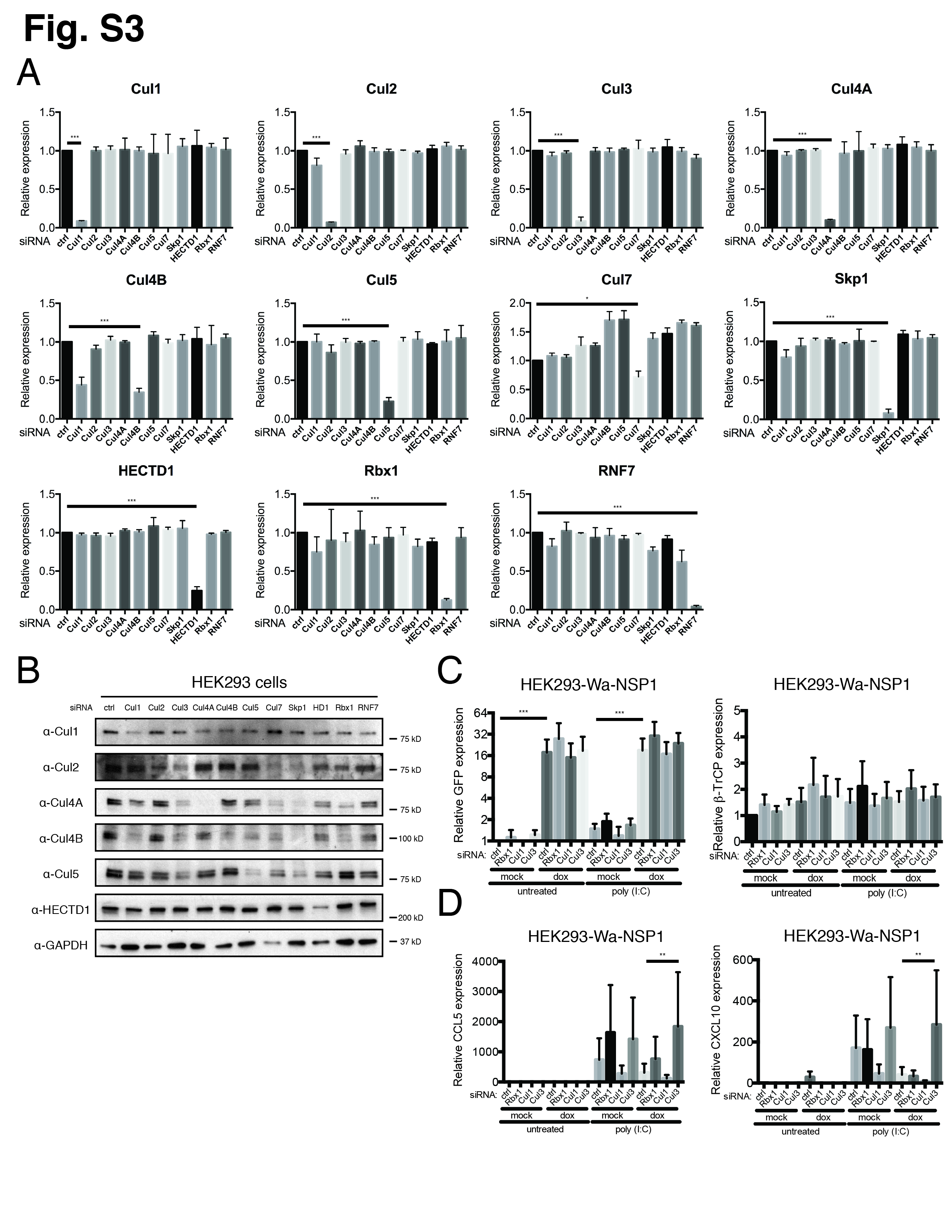

Supplement: S3 Fig — (A) HEK293 cells were transfected with indicated siRNA, and total RNA was extracted to measure by RT-qPCR the expression of indicated host genes, normalized to the levels of GAPDH. (B) Same experiment as in (A), except that the protein levels of indicated host genes were measured by western blot using indicated antibodies (HD: HECTD1). (C) HEK293 cells stably expressing Wa-NSP1 were transfected with indicated siRNA, treated with doxycycline, and stimulated with poly (I:C) (100 ng/ml) for 6 hr. RNA was extracted to measure by RT-qPCR the expression of GFP and β-TrCP, normalized to the levels of GAPDH. (D) Same experiment as in (C), except that the expression of CCL5 and CXCL10 was measured by RT-qPCR and normalized to GAPDH. In all figures, experiments were repeated at least three times. Data are represented as mean ± SEM. Statistical significance is determined by Student’s t test (*p≤0.05; **p≤0.01; ***p≤0.001). (TIF) [file ppat.1005929.s003.tif]

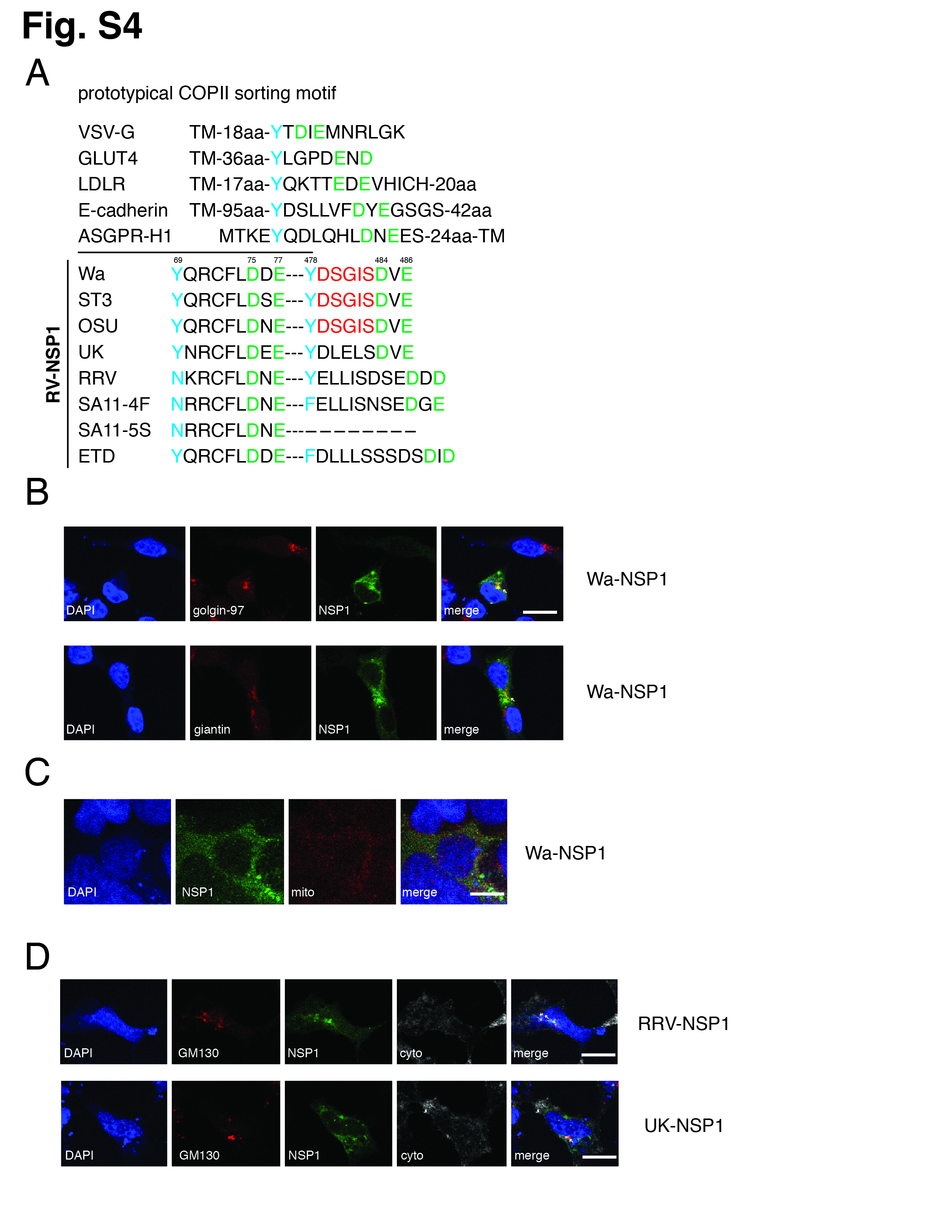

Supplement: S4 Fig — (A) Illustration of amino acid sequences within exemplary proteins that direct their incorporation into COPII-coated vesicles. VSV-G, vesicular stomatitis virus glycoprotein; GLUT4, glucose transporter type 4; LDLR, low-density lipoprotein receptor; ASGPR-H1, asialoglycoprotein receptor 1. Tyrosine (or other resides at the same location) and diacidic signal are highlighted in cyan and green respectively. Phosphodegron-like motif is highlighted in red. (B) HEK293 cells were transfected with pG-LAP6-Wa-NSP1 (green), and stained with Golgi markers golgin-97 (red, top panel) or giantin (red, bottom panel), and nucleus (DAPI, blue). Co-localization (yellow) is highlighted by white arrowheads. Panels are single z slices with a scale bar of 15 μm. (C) HEK293 cells were transfected with pG-LAP6-Wa-NSP1 (green), and analyzed by confocal microscopy for mitochondria (mito, red) and nucleus (DAPI, blue). Panels are single z slices with a scale bar of 10 μm. (D) HEK293 cells were transfected with pG-LAP6-RRV-NSP1 (green, top panel) or pG-LAP6-UK-NSP1 (green, bottom panel), and analyzed by confocal microscopy for the localization of Golgi (GM130, red), cytoskeleton (cyto, grey), and nucleus (DAPI, blue). Co-localization (yellow) is highlighted by white arrowheads. Panels are single z slices with a scale bar of 10 μm. In all figures, experiments were repeated at least three times. (TIF) [file ppat.1005929.s004.tif]

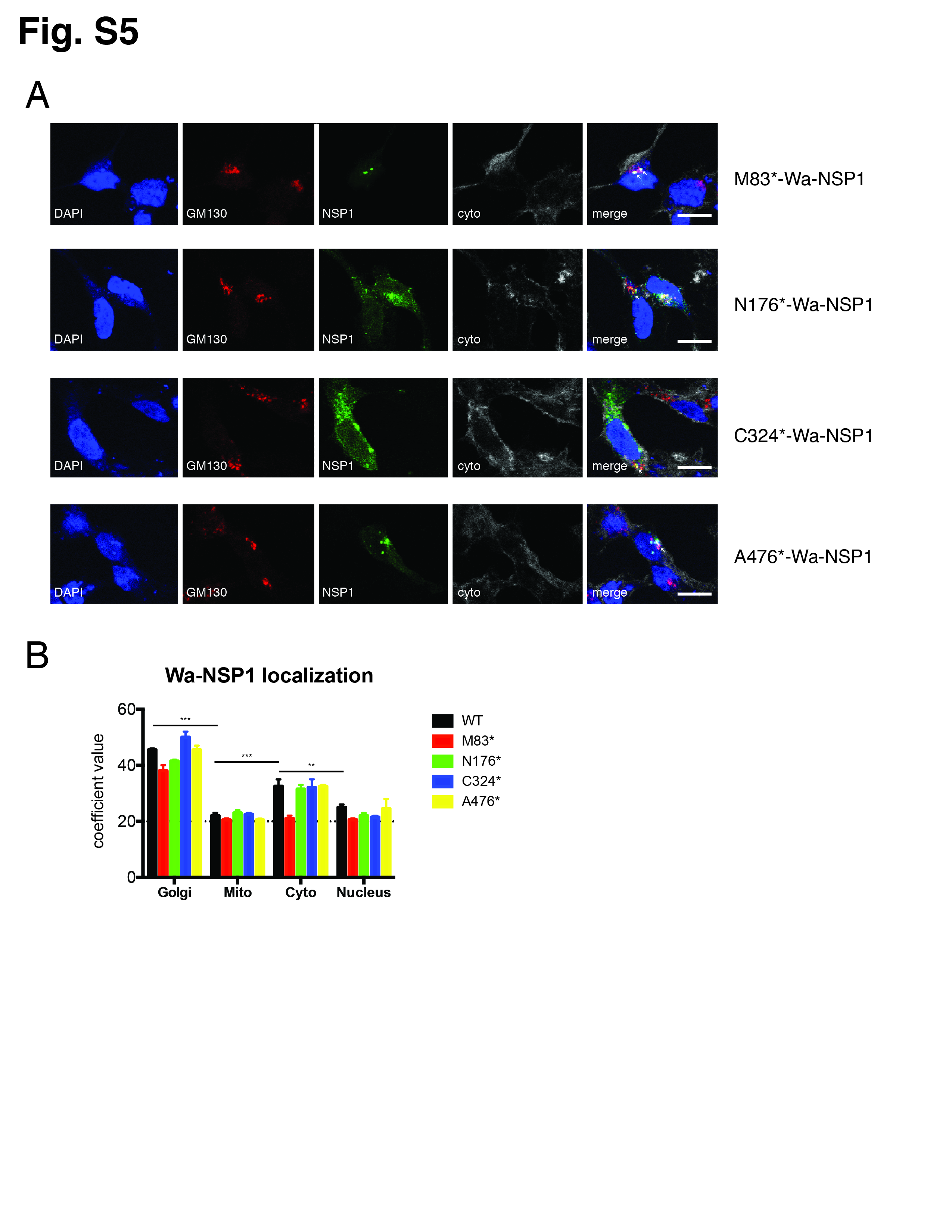

Supplement: S5 Fig — (A) HEK293 cells were transfected with pG-LAP6-Wa-NSP1 mutants M83*, L176*, C324*, and A476* (green), and analyzed by confocal microscopy for the localization of Golgi (GM130, red), cytoskeleton (cyto, grey), and nucleus (DAPI, blue). Co-localization (yellow) is highlighted by white arrowheads. Panels are single z slices with a scale bar of 10 μm. (B) The co-efficient value of co-localization of Wa-NSP1 protein (WT and mutants) and different cellular organelles was calculated in Volocity v5.2 on the basis of at least 20 micrographs. Co-localization of cytoskeleton and nucleus is set to be 20 (dotted line) and serves as the negative control. In all figures, experiments were repeated at least three times. Data are represented as mean ± SEM. Statistical significance is determined by Student’s t test (*p≤0.05; **p≤0.01; ***p≤0.001). (TIF) [file ppat.1005929.s005.tif]

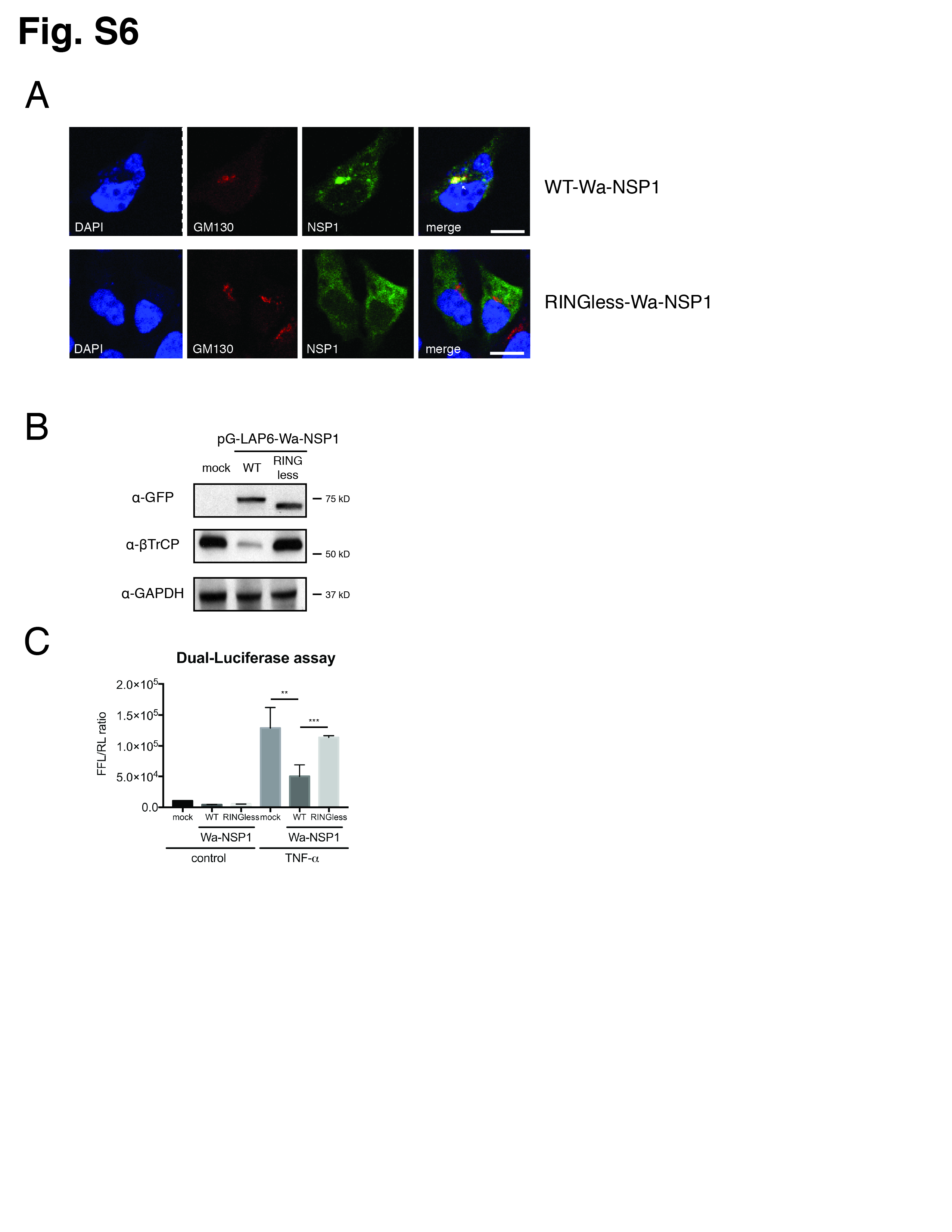

Supplement: S6 Fig — (A) HEK293 cells were transfected with pG-LAP6-Wa-NSP1 WT or mutant without RING-finger domain (RINGless, green), and analyzed by confocal microscopy for the localization of Golgi (GM130, red), and nucleus (DAPI, blue). Co-localization (yellow) is highlighted by white arrowheads. Panels are single z slices with a scale bar of 10 μm. (B) Lysates of HEK293 cells transfected with WT and RINGless Wa-NSP1s were analyzed by western blot using indicated antibodies. (C) HEK293 cells were co-transfected with PRDII-luc, pRL-TK and Wa-NSP1 mutants, stimulated with TNF-α (10 ng/ml) for 6 hr, and harvested for Dual-Glo luciferase assay. Arbitrary units were determined by the ratio of firefly luciferase (FFL) to the transfection control renilla luciferase (RL). In all figures, experiments were repeated at least three times. Data are represented as mean ± SEM. Statistical significance is determined by Student’s t test (*p≤0.05; **p≤0.01; ***p≤0.001). (TIF) [file ppat.1005929.s006.tif]

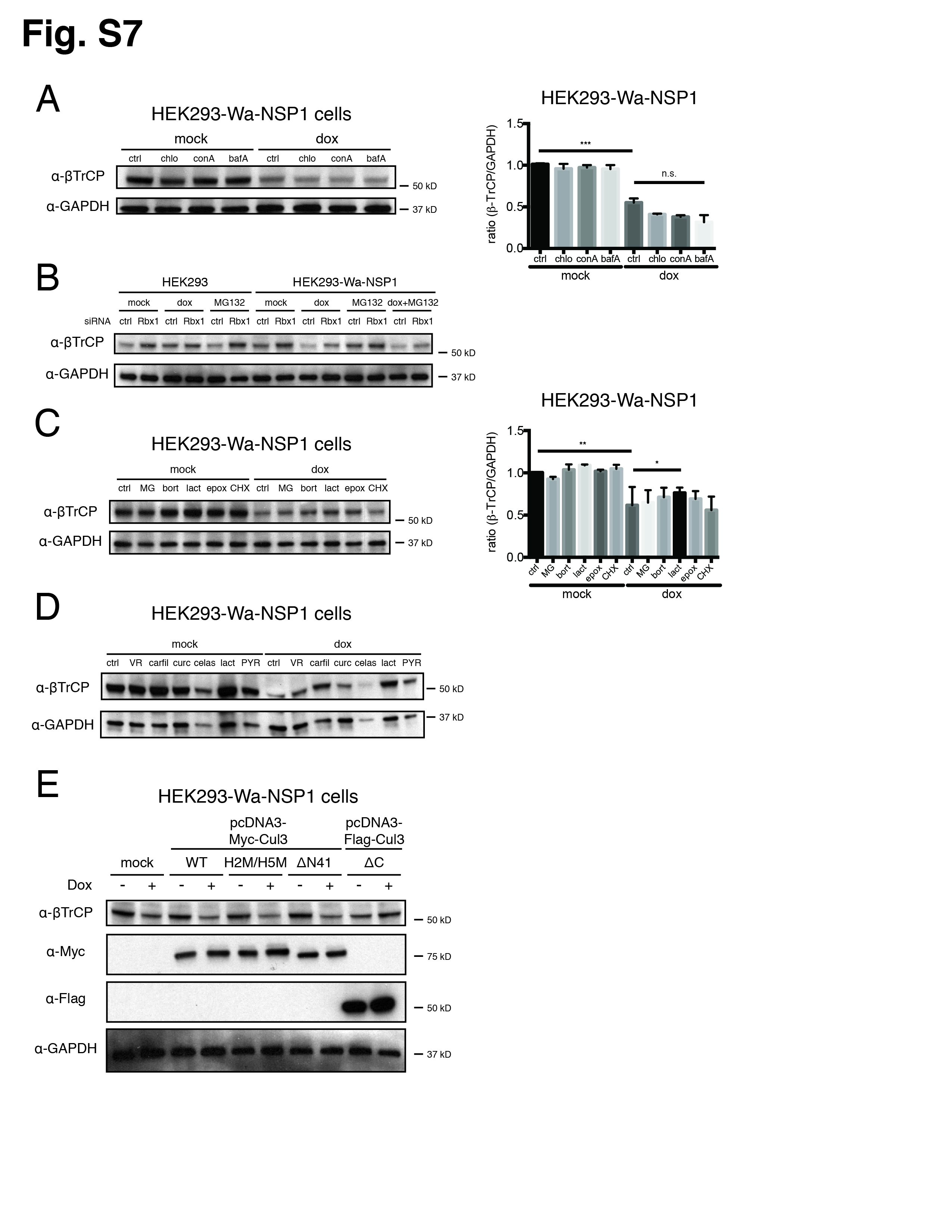

Supplement: S7 Fig — (A) Lysates of HEK293 cells stably expressing Wa-NSP1 were treated with doxycycline and indicated lysosome inhibitors, and analyzed by western blot using indicated antibodies. Blots were quantified and the level of β-TrCP is normalized to loading control GAPDH. The ratio of β-TrCP versus GAPDH in mock-treated cells is set to 1. (B) Wild-type HEK293 cells or HEK293 cells stably expressing Wa-NSP1 were transfected with indicated siRNA and treated with doxycycline and MG132. Lysates were harvested for western blot analysis using indicated antibodies. (C) Same experiment as in (A) except that proteasome and translation inhibitors were used (MG: MG132; bort: bortezomib; lact: lactacystin; epox: epoximicin; CHX: cycloheximide). (D) Same experiment as in (A) except that proteasome and E1 inhibitors were used (VR: VP23; carfil: carfilzomib; curc: curcumin; celas: celastrol; lact: lactacystin; PYR: PYR-41). (E) HEK293 cells stably expressing Wa-NSP1 were transfected with plasmids encoding WT or mutant Cul3, treated with doxycycline and harvested for western blot analysis using indicated antibodies. In all figures, experiments were repeated at least three times. Data are represented as mean ± SEM. Statistical significance is determined by Student’s t test (*p≤0.05; **p≤0.01; ***p≤0.001). (TIF) [file ppat.1005929.s007.tif]
