# Supplementary material for: Comparative Proteomics Reveals Strain-Specific β-TrCP Degradation via Rotavirus NSP1 Hijacking a Host Cullin-3-Rbx1 Complex
Source: PLoS Pathog. 2016 Oct 5;12(10):e1005929. doi: 10.1371/journal.ppat.1005929 (PMC5051689; doi:10.1371/journal.ppat.1005929)
Supplement: S7 Table — (DOCX) [file ppat.1005929.s014.docx]

| **SYBR Green QPCR Primers** |  |  |
| --- | --- | --- |
| BTRC | Forward | ACCAACATGGGCACATAAACTC |
|  | Reverse | TGGCATCCAGGTATGACAGAAT |
| CCL5 | Forward | CCAGCAGTCGTCTTTGTCAC |
|  | Reverse | CTCTGGGTTGGCACACACTT |
| CUL1 | Forward | GGTTCGCCGTGAATGTGAC |
|  | Reverse | CCCCAATTCCACGTAAGACTGT |
| CUL2 | Forward | CATGTTCGGCATTTGCATAAGAG |
|  | Reverse | GCACCCTTGCTGTATTCTTCC |
| CUL3 | Forward | GATGCACTGCCTTGACAAATCA |
|  | Reverse | CCTTGCTCCCTCAAATAGGAACT |
| CUL4A | Forward | ACCTCGCACAGATGTACCAG |
|  | Reverse | AGGTTGACGAACCGCTCATTC |
| CUL4B | Forward | ACTCCTCCTTTACAACCCAGG |
|  | Reverse | TCTTCGCATCAAACCCTACAAAC |
| CUL5 | Forward | TCCAAGATAGTGCAATGAAGCTG |
|  | Reverse | CTCTGTTGAATCCAAGTATGCCT |
| CUL7 | Forward | GGAGATGGAAACCGACGTGAA |
|  | Reverse | AGGAGGGATAGTGCCCACAC |
| CXCL10 | Forward | GTGGCATTCAAGGAGTACCTC |
|  | Reverse | TGATGGCCTTCGATTCTGGATT |
| GAPDH | Forward | GGAGCGAGATCCCTCCAAAAT |
|  | Reverse | GGCTGTTGTCATACTTCTCATGG |
| GFP | Forward | AAGCTGACCCTGAAGTTCATCTGC |
|  | Reverse | CTTGTAGTTGCCGTCGTCCTTGAA |
| HECTD1 | Forward | TATCTGCGGAATGTACCCGAA |
|  | Reverse | ACGAATGAAGGTAAGCACACAA |
| SKP1 | Forward | GACCATGTTGGAAGATTTGGGA |
|  | Reverse | TGCACCACTGAATGACCTTTT |
| RBX1 | Forward | TTGTGGTTGATAACTGTGCCAT |
|  | Reverse | GACGCCTGGTTAGCTTGACAT |
| RNF7 | Forward | TGGAAGACGGAGAGGAAACCT |
|  | Reverse | TCCCCAGACCACAACACAGT |
| RSAD2 | Forward | CAAGACCGGGGAGAATACCTG |
|  | Reverse | AACTCTACTTTGCAGAACCTCAC |
|  |  |  |
| **Dharmacon SMARTpool siRNA information:** |  |  |
| Non-targeting Control | D-001810-10 |  |
| CUL1 | L-004086-00 |  |
| CUL2 | L-007277-00 |  |
| CUL3 | L-010224-00 |  |
| CUL4A | L-012610-00 |  |
| CUL4B | L-017965-00 |  |
| CUL5 | L-019553-00 |  |
| CUL7 | L-017673-00 |  |
| FBXW11 | L-003490-00 |  |
| HECTD1 | L-007188-00 |  |
| RBX1 | L-004087-00 |  |
| RNF7 | L-006907-00 |  |
| SKP1 | L-003323-00 |  |
| ZBTB25 | L-019698-00 |  |
|  |  |  |
| **Cul3 CRISPR Knockout Information:** |  |  |
| sgRNA | Exon 7 | CTTACCTGGATATAGTCAAC |
| Sequencing primer | Forward | GTAGACCTTGGTTGCATGTAC |
| Sequencing primer | Reverse | CTACAGTATACAAACACTAAGGCATAC |
